# Positive Caricature Transcriptomic Effects Associated with Broad Genomic Aberrations in Colorectal Cancer

**DOI:** 10.1038/s41598-018-32884-3

**Published:** 2018-10-04

**Authors:** Daniele F. Condorelli, Giorgia Spampinato, Giovanna Valenti, Nicolò Musso, Sergio Castorina, Vincenza Barresi

**Affiliations:** 1 Department of Biomedical and Biotechnological Sciences, Section of Medical Biochemistry, University of Catania, Catania, (95123) Italy; 2 Department of Medical and Surgical Sciences and Advanced Technologies, University of Catania, Catania, (95123) Italy

## Abstract

We re-examined the correlation between Broad Genomic Aberrations (BGAs) and transcriptomic profiles in Colorectal Cancer (CRC). Two types of BGAs have been examined: Broad Copy-Number Abnormal regions (BCNAs), distinguished in gain- and loss-type, and Copy-Neutral Loss of Heterozygosities (CNLOHs). Transcripts are classified as “OverT” or “UnderT” if overexpressed or underexpressed comparing CRCs bearing a specific BGA to CRCs not bearing it and as “UpT” or “DownT” if upregulated or downregulated in cancer compared to normal tissue. BGA-associated effects were evaluated by changes in the “Chromosomal Distribution Index” (CDI) of different transcript classes. Data show that UpT are more sensitive than DownT to BCNA-associated gene dosage effects. “Over-UpT” genes are upregulated in cancer and further overexpressed by gene dosage, defining the so called “*positive caricature transcriptomic effect*”. When Over-UpT genes are ranked according to overexpression, top positions are occupied by genes implicated at the functional and therapeutic level in CRC. We show that cancer-upregulated transcripts are sensitive markers of BCNA-induced effects and suggest that analysis of positive caricature transcriptomic effects can provide clues toward the identification of BCNA-associated cancer driver genes.

## Introduction

The transformation of a normal cell into a cancer cell can be described as a pathological evolutionary process based on the generation and selection of cellular phenotypic changes providing growth advantages to clonal cell populations^1,2^. Such growth-advantageous and selectable changes are produced by *driver mutations* that can be classified, according to the type of DNA modifications, in single nucleotide substitutions, small indels, gene fusions, copy number abnormalities and epigenetic modifications. However, the same process can randomly generate *passenger mutations*, that are simply co-selected with driver mutations. In terms of a cancer evolutionary process the driver mutations can contribute to positive selection, while the passenger ones can be considered neutral. One of the key point in molecular oncology research is to distinguish between driver and passenger mutations and to define their contribution to the cancer phenotype or vulnerability^3^.

The actual knowledge on DNA-encoded biological information provides the conceptual framework necessary to evaluate the functional impact of single-gene mutations, such as point mutations, small indels and gene fusions. Gain- or loss-of-function mutations in single genes can be connected to the generation of selectable features leading to the cancer phenotype and these concepts have been condensed in the definition of oncogenes (OGs) and tumor suppressor genes (TSGs). The pathogenetic role of some types of large structural chromosomal aberrations, such as translocations, in cancer is well-established and is mainly due to the formation of gene fusions at the breakpoints^4^. On the other hand, the distinction between driver and passenger mutations and the connection of driver mutations to selectable functional properties is much more difficult in the case of Broad Genomic Aberrations (BGAs) that produce a gene copy number change, a common type of mutational event in cancer that involves of a large number of adjacent genes in a single step^5^. The subgroup of BGAs, that are characterized by losses or gains of large chromosomal DNA segments or whole chromosomes (aneuploidy) in cancer cells, are also known as somatic Broad Copy Number Abnormalities (BCNAs). Indeed, the majority of BCNAs are represented by whole-chromosome or chromosomal arm gains or losses i.e. monosomy, trisomy, or tetrasomy of whole chromosomes or chromosomal arms^6–9^.

A large fraction of common solid tumors, such as colorectal cancer, are characterized by chromosomal instability defined by an increased rate of BGAs acquisition, including many BCNAs. A first challenge is to distinguish between driver and passenger BCNAs: the high recurrence rate and functional and clinical data suggest that some BCNAs can be considered as driver mutational events^9^. A second challenge is to understand which gene or genes contained in the BCNA chromosomal region are responsible for the driver properties of the mutational event.

A common interpretation of the selectability of BCNAs (driver properties) is still based on a single-gene point of view. For instance, in the context of the classical two-hit mechanism for inactivation of TSG, providing that the first allele has been inactivated by a single-gene mutational events (inactivating point mutation or small focal deletion), the remaining functioning allele can be removed by a broad genomic loss (for instance a chromosome monosomy). Indeed, the probability that a second hit is represented by a broad genomic loss is very high in comparison to the probability of a second focal event hitting the corresponding locus in the homologous chromosome. Moreover, a single mutational event, such as a broad loss, can represent the second hit for more mutated TSGs, thus producing the simultaneous biallelic inactivation of multiple TSGs (multi-gene second hit). Along this line of reasoning, the accompanying hemizygous losses of several genes contained in the same genomic segment can be considered as passenger mutations, i.e. neutral for the evolution towards a cancer phenotype. In contrast with this simple view, it is conceivable that the simultaneous loss or gain of several genes contained in broad deleted or amplified chromosomal regions can also exert positive (driver) or negative (deleterious) effects for cancer phenotype selection^5,10^.

For a better understanding of the cancer driver effects of BCNAs, it is relevant the distinction between two types of TSGs based on the property of haploinsufficiency^11^. The term haploinsufficiency indicates that the monoallelic inactivation of a gene is able to generate selectable phenotypic modifications. TSGs that do not show haploinsufficiency (haplosufficient or recessive TSGs) need a classical second hit (biallelic inactivation) in order to produce cancer driver effects. On the contrary haploinsufficient TSGs could exert driver effects independently by a second hit. It has been suggested that a large majority of TSGs involved in sporadic cancer are haploinsufficient^11^. However, single-gene haploinsufficiency is likely to be a low-potency effect and the synergistic haploinsufficiency of multiple genes (*cumulative or multi-gene haploinsufficiency*) might be necessary to generate a relevant driver effects^11^. Similar reasoning can be applied to the so called “triplosensitivity”, indicating the ability of an additional copy of a gene to contribute to the cancer phenotype. In the latter case the increased copy number of multiple OGs, or other form of cancer promoting genes, can play synergistic driver effects (*cumulative or multi-gene triplosensitivity)*^11^, independently by the presence of activating single-gene mutations.

A combination of the above mentioned mechanisms (single- or multi-gene second-hit, multi-gene haploinsufficiency and triplosensitivity) can represent the basis for a general theory explaining the non-random chromosomal distribution of recurrent BCNAs in cancer. However, the relative contributions of the different mechanisms and several quantitative aspects of this general theory need to be clarified and proved. Moreover, it is not clear to what extent the same mechanisms apply to a different type of BGA, called copy-neutral loss of heterozygosity (CNLOH), that is frequent in cancer but does not imply any change in gene copy number^12–23^. An important functional property of CNLOH is the ability to convert monoallelic into biallelic mutations of TSGs during cancer progression^13,18,19,24^. Therefore, the single- or multi-gene two-hit mechanism may contribute to the selection of CNLOHs during cancer evolution. Another possible mechanism is the duplication of activating oncogenic mutations^25^. However, our knowledge on putative cancer driver effects of CNLOH is still limited and more details are needed.

The easiest molecular mechanism that links cumulative haploinsufficiency or triplosensitivity to the cancer phenotype is the change of transcript level correlated to gene dosage (gene dosage transcription effect). With this in mind we decided to re-examine some aspects of the correlation between copy number changes in large chromosomal segments (BCNAs) and transcriptomic changes in colorectal cancer (CRC). Indeed, it has been repeatedly shown that transcript levels of a significant proportion of genes are correlated to gene copy number in cancer genomes^5,6,26–35^. Moreover, since it has been reported that a subgroup of genes in CNLOH region change their expression by gene dosage independent mechanisms^17^, we also examined the association between whole chromosome or arm-level CNLOH and transcriptome changes.

Since early application of dedicated statistical analysis to SNP array data it was clear that significant aberrations in cancer genome fall into two types: focal and broad (whole chromosome or arm-level aberrations)^6–9^. These studies have shown that somatic CNA frequencies across different cancers are inversely proportional to CNA lengths, with the exception of CNAs near the size of a chromosome arm or whole chromosome (called BCNAs in the present work), which are very frequent^6–9^. Beroukhim *et al*.^6^ have already suggested that focal and broad CNAs can have very different consequences for tumor biology. However, most of the following research has been focused on focal CNAs because of the difficulties to identify driver genes in broader regions^7,8,33,36,37^. In the present work we concentrate our attention on broad regions of chromosomal aberration, using a procedure that allows the identification of BCNAs even in the presence of superimposed focal events or artifacts due to cancer heterogeneity^38^. The main hypothesis behind the choice to analyze BCNAs is that the mechanisms providing cancer growth advantage are, at least in part, different between focal and broad chromosomal aberrations^5^. As extensively described it is possible that recurrent BCNAs are acting through the mechanisms of multi-gene triplosensitivity or haploinsufficiency and that the involved multigene networks are different from those of focal aberrations^6^. Moreover, in contrast to focal CNAs, few arm-level CNAs reach high amplitude changes in copy number^7^, and this feature should be taken into consideration in hypothesis regarding BCNA role in cancer progression.

One of the most used parameter in the integrative analysis of chromosomal abnormalities and gene expression is the gene-by-gene correlation index of gene copy number with transcript level^35^. However, this strategy is better suited for focally high-level amplified genes and might underestimate the contribution of genes located in large low-level gain regions, such as trisomic chromosomes. It has been also suggested that this approach might introduce a bias toward genes whose expression is almost exclusively modified by the BCNA-associated gene dosage effect, while neglecting those genes that are also strongly regulated by different regulatory processes^34^.

In the present work the influence of BGAs, both copy number abnormal and copy number neutral, on transcriptome profile was investigated by SNP- and transcriptome-arrays in our series of **c**olo**r**ectal **c**ancer (CRC) samples adopting the following strategy: (1) identification of arm-level or entire chromosome BGAs by robust methods based on log_2_ratio, size thresholds and allele difference^19,38^, (2) generation of different CRC groups based on the presence of a selected BGA in the study group and its absence in the corresponding control groups, (3) identification of genes that are differentially expressed (at the transcript level) between selected CRC group and corresponding control CRC group (called OverT or UnderT genes) or between CRC group and corresponding normal colonic tissues (called UpT and DownT genes), (4) evaluation of the contribution of genes located in a specific BGA region (BGA-genes) to a given transcript class by a novel parameter called “Chromosomal Distribution Index” (CDI).

Results show that cancer-upregulated transcripts are sensitive markers of BCNA-dosage effects and reveal a preferential involvement of gain BCNAs in a type of transcriptomic change denominated positive caricature transcriptomic effect.

## Results

### Chromosomal distribution of Broad Copy Number Abnormalities (BCNAs) and Somatic Broad Copy-Neutral Loss-Of-Heterozygosities (SB-CNLOHs) in CRC

**Figure 1:**
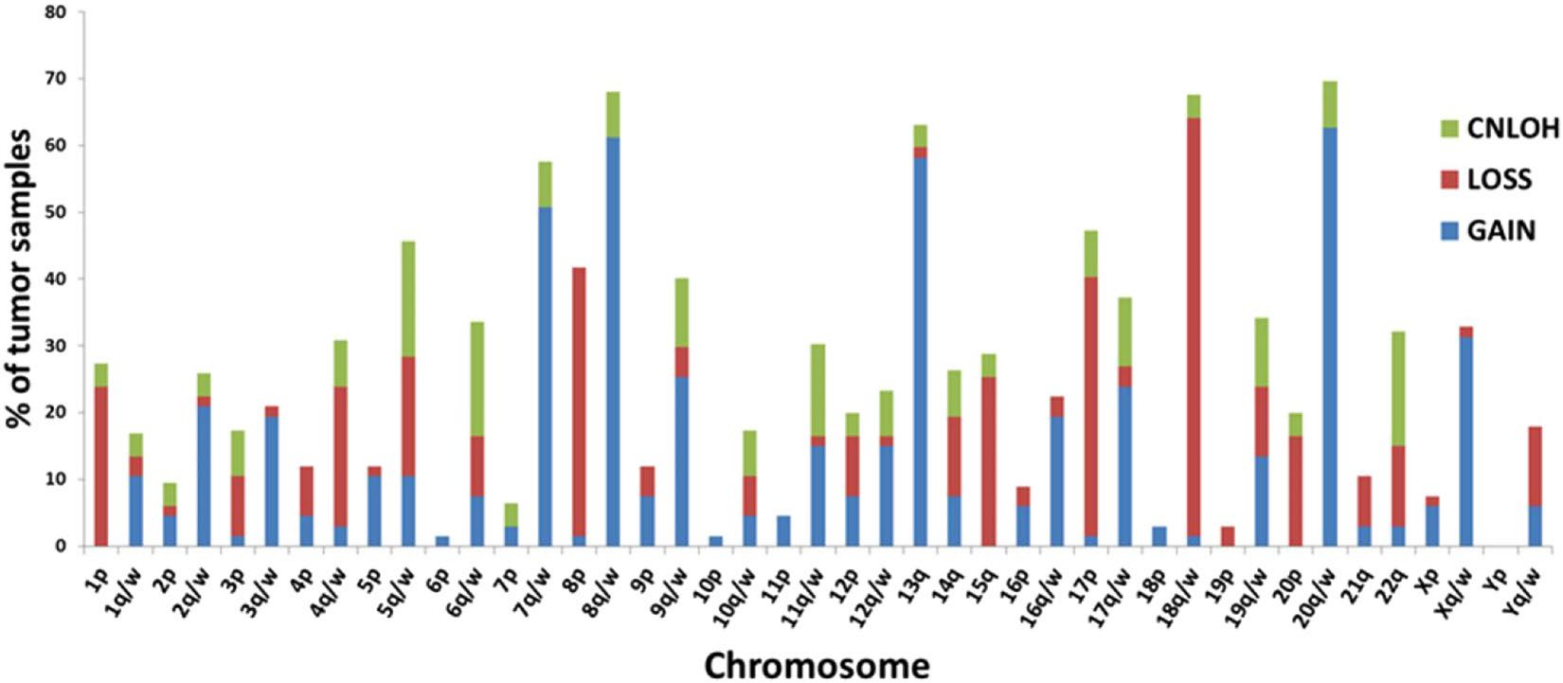
Chromosomal distribution of BCNAs and SB-CNLOHs (>3 Mb) in CRC. Results are expressed as percentage of tumor samples bearing BCNAs or SB-CNLOH in p arm, q arm or whole chromosome (w). Results of q arm and whole chromosomes have been combined.

SNP array analysis provides data on Copy Number Abnormalities (CNAs), at a lower limit of tens of kb. Among CNAs we selected the Broad Copy Number Abnormalities (BCNAs) defined as chromosomal aberrations that involve more than 25% of a chromosomal arm (p or q) or a whole chromosome (w). BCNAs can be distinguished in broad losses and broad gains. As previously reported^38^ broad gains and broad losses show a different distribution (Fig. 1). A high frequency (≥25% of the analyzed CRC samples) of broad gains can be observed in 20q/w (63%), 8q/w (61%), 13 (58%), 7q/w (51%), Xq/w (31%), 9q/w (25%) and a high frequency of broad losses in 18q/w (63%), 8p (40%), 17p (39%), 15q (25%). As expected the frequencies of gains and losses in different chromosomes show an inverse relationship.

The same SNP array analysis allows identification of CNLOH regions. The parameter “allele difference” (difference between allele A and allele B signal, each standardized with respect to their median values in the reference population) was used for visualization of CNLOH regions^18,23^. An analysis of somatic CNLOH regions larger than 3 Mb, called somatic broad CNLOH (SB-CNLOH), was performed in 29 tumor/normal tissue couples. The somatic nature of the chromosomal aberration was established in those samples by comparison with the corresponding normal tissue. Chromosomal distribution of SB-CNLOHs is non-random but show a pattern different from both gains and losses. In general, the frequency of SB-CNLOH is lower than that of BCNAs. The chromosomes that show the highest frequency of SB-CNLOHs, such as 22q (17%), 6q/w (17%), 5q/w (17%), have only low or intermediate frequencies of BCNAs (Fig. 1) and SB-CNLOHs are the predominant chromosomal aberrations in Chr6 and Chr22q. Moreover, CNLOH regions in Chr6 and 22q span the entire chromosome or the vast majority of the q arm.

### Somatic BGAs and Transcriptome Analysis

In order to distinguish the transcriptional effects associated to BGAs, both gene-dosage dependent and independent effects, from those related to other forms of cancer-associated transcriptional dysregulations, we organized CRC samples in groups bearing a specific somatic BGA in a selected chromosome called “*Selected CRC”* group. Each selected CRC group was compared with a corresponding “*Control CRC*” group composed of tumors lacking any aberrations on the chosen chromosome. Five selected CRC groups were characterized by broad gains in Chr20, Chr8q, Chr13, Chr7, Chr9 and another two selected CRC groups by broad losses in Chr8p and Chr18. Moreover, we analyzed two “selected CRC” groups characterized by SB-CNLOH in Chr6 and Chr22 that, by definition, do not bear any copy number changes in the selected BGA regions. In Supplementary Table S1 we report some clinical (age, gender, anatomical site, tumor stage) and molecular features (MicroSatellite Instability “MSI” status, activating KRAS mutations, Consensus Molecular Subtypes “CMS” according to Guinney *et al*.^39^). As expected, CMS2 is enriched in the selected gain and loss CRC groups, in agreement with the fact that this molecular subtype displays higher chromosomal instability, measured by somatic CNA count^39^. On the contrary CMS1, that displays an almost normal karyotype, and CMS3, that bears fewer somatic CNAs in comparison to CMS2, are enriched in the corresponding control CRC groups. The enrichment of CMS2 is not observed in CNLOH selected CRC groups. MSI tumors are enriched in the control CRC groups (with the exception of Chr8q-gain), in agreement with the fact that MSI tumors display an almost normal karyotype. As expected, they represent a minor fraction of control CRC groups (9–26%).

The frequencies of somatic genomic aberrations in “Selected CRC” and “Control CRC” groups are reported in Supplementary Table S2. For instance, all tumors (100%) in the Chr7-gain group show broad gains in Chr7q or whole Chr7, while the control group does not bear any type of broad aberrations in Chr7 (or isolated focal aberrations as defined in Barresi *et al*.^38^). Somatic genomic aberrations in other chromosomes were distributed among the selected and control groups. Indeed, some genomic aberrations show a preferential association: for instance, Chr7-gains are associated to gains of Chr8q (68%), Chr13 (73%), Chr20 (86%) and ChrX (55%). The selection did not take into account the presence of aberrations on the p arm and the same strategy was used for groups based on other chromosomes, except for Chr8. A separate analysis of p and q arm of Chr8 was performed (see Supplementary Table S2), since deletions of p arm of Chr8 were often associated to duplications of the q arm.

**Figure 2:**
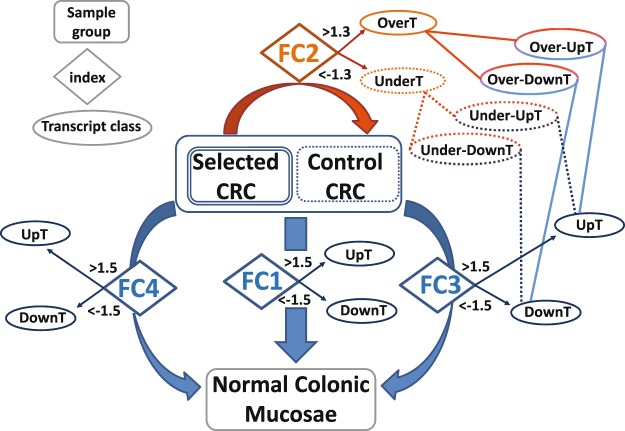
Sample groups, Fold-Changes (FC) and transcript classes. Transcript classes defined on the basis of four indices (FC1, FC2, FC3, FC4), three CRC groups (All CRC, Control CRC, Selected CRC) and one Normal Colonic Mucosae group (Normal). CRCs with specific BGA form a “Selected CRC” group while CRC without specific BGA form a “Control CRC” group. Fold-changes (FC) are obtained by comparisons of RMA averages between different groups: FC1 between “all CRC” and “Normal”, FC2 between “Selected CRCs” and “Control CRCs”, FC3 between “Control CRC” and “Normal”, FC4 between “Selected CRC” and “Normal”.

Then, transcriptome analysis has been performed and comparisons between sample groups are summarized in Fig. 2 and Supplementary Table S3. Four different *indices* were used in order to estimate transcript levels in CRC and to define different transcript classes: (a) linear fold-changes obtained comparing all CRC samples vs matched normal colonic mucosae (denominated FC1), (b) linear fold-changes obtained comparing CRCs bearing a specific BGA (Selected CRC group) to CRCs not bearing a specific BGA (Control CRC group) (denominated FC2), (c) linear fold-changes obtained comparing “Control CRC” group to normal colonic mucosae (denominated FC3), (d) linear fold-changes obtained comparing “Selected CRC” group to normal colonic mucosae (denominated FC4) (Fig. 2 and Supplementary Table S3).

In order to analyze transcripts that contribute significantly to variability among groups we selected transcripts that show statistically significant changes among selected CRC group, control CRC group and matched normal tissues (multiple comparisons) at a False Discovery Rate (FDR) adjusted p-value < 0.05^40^, denominating them as Variable Transcripts (VT). About 83% of transcripts transcribed on each chromosome were VT. Variable Transcripts are then grouped in different “transcript classes” according to cutoff values of the previously defined indices (Fig. 2).

The number of transcripts belonging to a specific “transcript class” and transcribed in a specific chromosomal region (chromosome or chromosome arm) is reported as percentage of the total number of transcripts belonging to that class in the entire genome, thus obtaining the chromosomal distribution of a transcript class among each of the 24 chromosome types. We called this parameter “Chromosomal Distribution Index” (CDI) and calculated it with the following formula:

$${\rm{CDI}}\,{\rm{of}}\,{\rm{chromosomal}}\,{\rm{region}}\,n={x}_{n}\,\ast \frac{1}{{\sum }_{i=1}^{T}{x}_{i}}\ast \,100$$

where *x*_*n*_ is the number of transcript belonging to a transcript class transcribed in the *n-th* chromosomal region and T is the total number of chromosomal regions subdividing the entire genome. The CDI of VT is strictly correlated to that of all analyzed transcripts, “all T” (Pearson’s r index = 0.99), thus showing that the chromosomal distribution of VT is simply dependent on the number of transcripts synthesized on each chromosomes. On the contrary significant deviations from “all T CDI” were observed when other transcript classes were analyzed. The difference between CDI of a specific transcript class and “all T CDI” on a selected BCNA chromosome provides an estimate of the sensitivity of such transcript class to BCNA-induced transcriptional cis-effects.

In the present paper we use the terms “Overexpressed Transcripts” (OverT) to indicate those “VT” that show a FC2 >1.3 and “Underexpressed Transcripts” (UnderT) to indicate those VT that show a FC2 < −1.3 by comparing “Selected CRCs” versus “Control CRCs” (Fig. 2).

**Figure 3:**
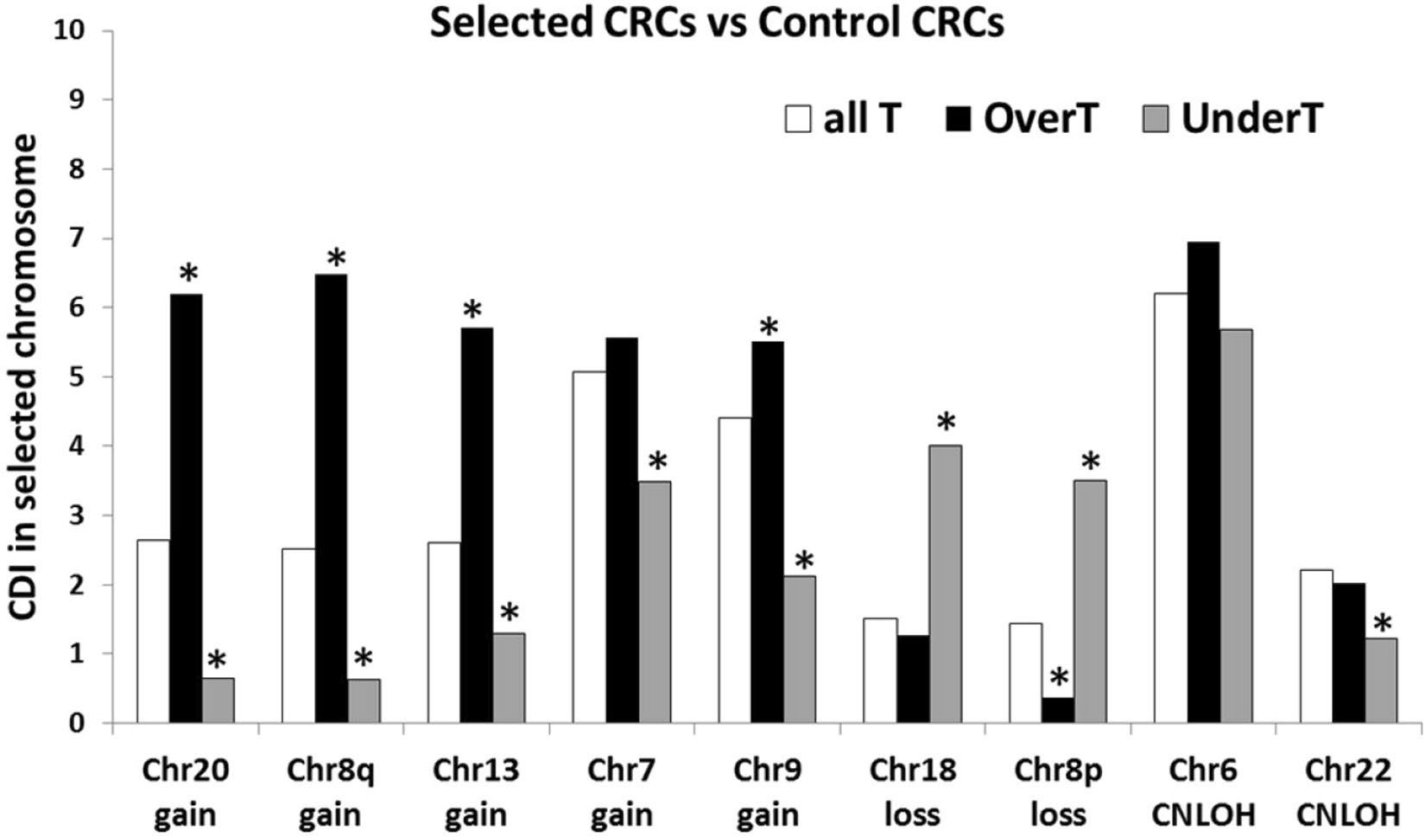
“OverT” and “UnderT” classes. CDI of OverT and UnderT in selected chromosomes of each Selected CRC group. CDI of all transcripts analyzed in HTA (All T) is also reported for comparison. *p < 0.01 comparing the selected chromosome versus all other chromosomes by chi-squared test.

Figure 3 summarizes the CDI values obtained for OverT and UnderT for each selected chromosome in all selected CRC groups (results obtained for every chromosome in all groups are reported in Supplementary Fig. S1). By comparison with CDI of all T, an increase of OverT CDI and a corresponding decrease of UnderT CDI in selected aberrant chromosomes is detected in “gain-groups”. An opposite trend is present in “loss-groups”: a significant increase of UnderT CDI is observed in “Chr18-loss” and “Chr8p-loss” groups, while a significant decrease of OverT CDI is observed only in “Chr8p-loss” group (Fig. 3). No significant difference is observed in the CNLOH groups, except for a significant decrease of UnderT CDI in Chr22.

Other types of transcript classes are based on the detection of differentially expressed transcripts between CRC and normal colonic tissue. In order to distinguish these transcripts from OverT and UnderT we use the terms “Upregulated Transcripts” (UpT) to indicate those VT that show a FC3 or FC4 >1.5 and “Downregulated Transcripts” (DownT) for those VT that show a FC3 or FC4 <−1.5 by comparing CRC versus normal colonic tissue (Fig. 2). It is obvious that “upregulated” and “overexpressed” (or “downregulated” and “underexpressed”) are usually considered as synonyms, but, for the purpose of this work, we are attributing a different meaning to these terms.

**Figure 4:**
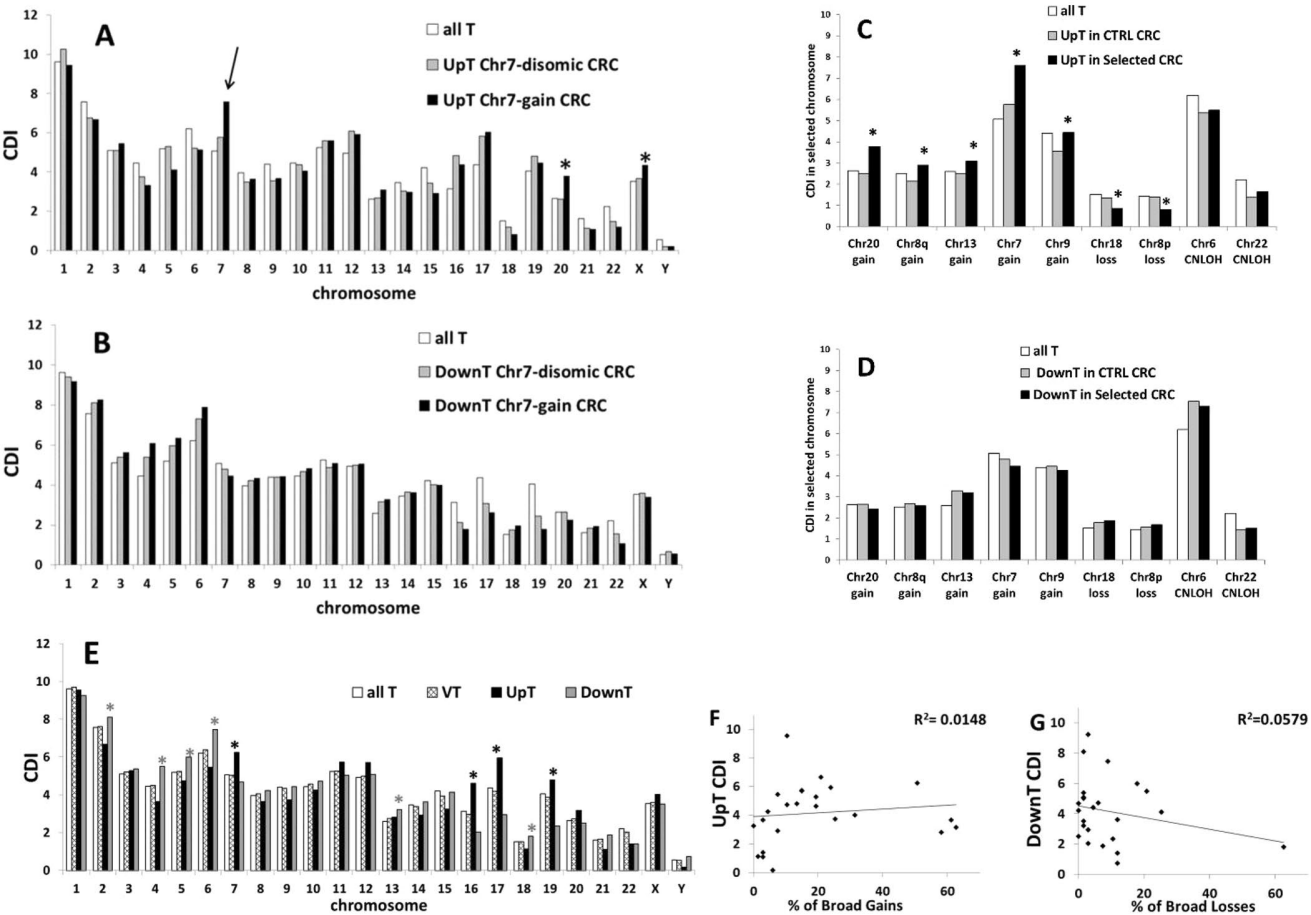
“UpT” and “DownT” classes. CDI of UpT (**A**) or DownT (**B**) in Chr7-gain group and its control group (Chr7-disomic group). Arrow indicates a significant increase of UpT in Chr7 in the Chr7-gain CRC group (p < 0.05 comparing Chr7-gain CRC vs Chr7-disomic CRC by chi-squared test). A significant increase of UpT in Chr20 and ChrX can be also observed in A (*p < 0.05). CDI of UpT (**C**) and DownT (**D**) in selected chromosomes of each selected CRC group and its corresponding control (CTRL) CRC group. Chromosomal distribution of all transcripts (all T) is also reported for comparison. *p < 0.05 comparing selected CRC vs CTRL CRC by chi-squared test. (**E**) CDI of all T (all transcripts analyzed in HTA array), VT (all statically significant variable transcripts, FDR < 0.05), UpT (statistically significant VT that show a FC1 > 1.5 in comparison to normal tissue) and DownT (statistically significant VT that show a FC1 < −1.5 in comparison to normal tissue). Black star indicates significant increase of UpT in a specific chromosome (p < 0.002 by chi-squared test in comparison to all other chromosomes); grey star indicates significant increase of DownT in a specific chromosome (p < 0.002 by chi-squared test). (**F**) Correlation analysis between percentage of UpT in each chromosome and the frequency of broad gains in the same chromosome, expressed a percentage of cancer samples bearing broad gains for that given chromosome. (**G**) Correlation analysis between percentage of DownT in each chromosome and the frequency of broad losses in the same chromosome, expressed a percentage of cancer samples bearing broad losses for that given chromosome. Squared Pearson’s correlation coefficients (R^2^) are reported in each plot in F and G.

Figure 4 shows a comparison of the chromosomal distribution of UpT CDI (Fig. 4A) or DownT CDI (Fig. 4B) in Chr7-gain group and its control group (Chr7-disomic group). A clear increase of UpT CDI on Chr7 can be observed in Chr7-gain group in comparison to Chr7-disomic group. A similar increase can be observed in Chr20 and ChrX, in agreement with a partial enrichment of gains in these chromosomes in the Chr7-gain group (Supplementary Table S2).

Figure 4C,D summarizes the results obtained for each selected chromosome in all “selected CRC” groups. A significant increase of UpT CDI in selected aberrant chromosomes is detected in all “gain” groups, while a decrease can be observed in the two “loss” groups (p < 0.05 comparing “selected CRC” vs “control CRC” by chi squared test, Fig. 4C). As expected no significant difference was observed in the CNLOH groups. These results confirm that a significant fraction of transcripts on BCNA- chromosomes follows a gene dosage transcriptional rule and that the BCNA-associated effect allows a group of transcripts to exceed the threshold to be classified as UpT in the “gain” groups or decreases the gene expression below such threshold in the “loss” groups. On the contrary, DownT CDI in aberrant chromosomes are not significantly different between control CRC group and selected CRC group (Fig. 4D), suggesting a lower sensitivity in the detection of BCNA-associated effects in the DownT class.

We also analyzed the entire collection of CRC samples as a single group in comparison to matched normal colonic mucosae (FC1 as shown in Fig. 2 and Supplementary Table S3), in order to test the correlation between CDI values of UpT (FC1 >1.5) and DownT (FC1 <−1.5) in each chromosome and the frequencies of the corresponding aberrant chromosomes. As a first step we identified VT between all CRCs and matched normal tissues (pairwise comparison) at a false discovery rate (FDR) adjusted p-value < 0.05. The CDI of VT among the various chromosomes is strictly correlated to the CDI of all T (Fig. 4E, Pearson’s r index = 0.99), while the CDI of UpT and DownT show some relevant deviations from all T distribution. For instance, a highly significant increase of UpT CDI can be observed in Chr7, Chr16, Chr17 and Chr19 (p < 0.002 in comparison to all other chromosomes by chi squared test), while an increase of DownT CDI is detected in Chr2, Chr4, Chr5, Chr6, Chr13, Chr18, Chr21 (p < 0.002). It should be noted that chromosomes, such as Chr13, Chr20 and Chr2, that are frequently trisomic in CRC, do not show an enrichment in UpT. On the contrary, Chr13 and Chr2 show an increase of DownT CDI. Moreover, the chromosomal distributions of UpT and DownT are not significantly correlated to the frequencies of gains or losses detected at each chromosome (Fig. 4F,G). Therefore, the presence of a large number of upregulated or downregulated transcripts on a chromosome is not predictive of the frequency of gain or loss genomic aberrations.

In a subsequent analysis we calculated the CDI of the other four classes of transcripts defined by a combination of FC2 and FC3 indices, as indicated in Fig. 2 and Supplementary Table S3: Over-UpT, Under-UpT, Over-DownT, and Under-DownT. It should be noted that UpT or DownT in this subsection refer to genes that are differentially expressed when comparing control CRCs to normal tissue (comparison evaluated by FC3 as shown in Fig. 2 and Supplementary Table S3), while OverT and UnderT genes refer to the comparison of selected BGA-bearing CRCs versus control CRCs (comparison evaluated by FC2 as shown in Fig. 2 and Supplementary Table S3). Since the overexpression of UpT (Over-UpT) can be viewed as an exaggeration of a distinctive transcriptional feature of tumors we defined such effect as a “caricature transcriptomic effect”. Indeed, a caricature is a representation, usually pictorial or literary, in which the subject’s distinctive features or peculiarities are exaggerated to produce grotesque effect (http://www.thefreedictionary.com). In this context the underexpression of UpT (Under-UpT) is defined as an “anticaricature effect”, since it produces an attenuation of a distinctive feature. Therefore, similar caricature and anticaricature effects on DownT are represented respectively by Under-DownT and Over-DownT. Moreover, caricature and anticaricature effects on UpT are collectively called “positive effects”, while caricature and anticaricature effects on DownT are denominated “negative effects”. We evaluated the contribution of transcripts synthesized in the BGA chromosomal regions to such caricature and anticaricature effects, by analyzing the CDI of the four transcript classes.

**Figure 5:**
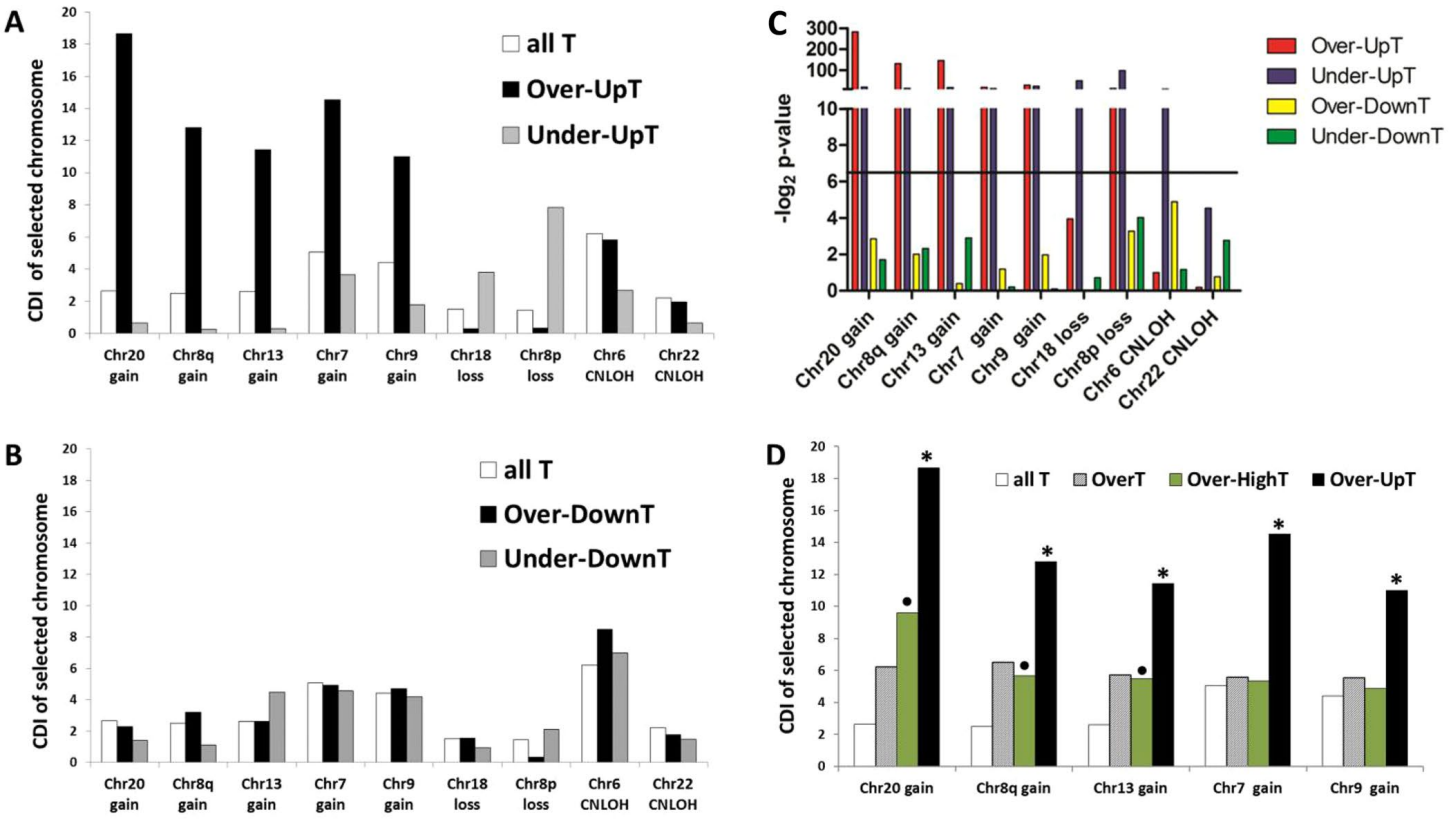
Caricature and Anticaricature Transcriptomic Effects. CDI of Over-UpT and Under-UpT (**A**) and Under-DownT and Over-DownT (**B**) in selected chromosomes of each selected CRC group. Chromosomal distribution of all transcripts analyzed in HTA (all T) is also reported for comparison. (**C**) negative log_2_ of p-values calculated by chi-squared test comparing the selected chromosome versus all other chromosomes; a horizontal black line indicates a p-value = 0.01. (**D**) CDI of Over-UpT and Over-HighT in selected chromosomes of gain-CRC group. Chromosomal distribution of all transcripts (all T) and OverT is also reported for comparison. *p < 0.001 comparing CDI of Over-UpT vs Over-HighT or OverT by chi-squared test. •p < 0.01 comparing CDI of Over-HighT vs AllT.

Figure 5A–C show a comparison of the CDI values of the four transcript classes with “all T CDI” in each selected aberrant chromosome and in all selected CRC groups. A sharp and significant increase of “Over-UpT CDI” is observed in all the gain-chromosomes, while a decrease is detected in the loss-chromosomes and no change in the CNLOH-ones (Fig. 5A and C). As far as anticaricature effects are concerned, the more evident result is an increased “Under-UpT CDI” in loss-chromosomes (Chr8p-loss and Chr18-loss group, Fig. 5A and C). A decreased “Under-UpT CDI” is also detected in the CNLOH groups (Fig. 5A and C). Analysis of caricature and anticaricature effects on DownT (negative effects) does not reveal significant or consistent changes of CDI among groups bearing the same type of abnormality (Fig. 5B and C). In conclusion, chromosomal distribution reveals that a subgroup of transcripts synthesized in gain-type chromosomal regions largely contribute to the global caricature effect on UpT (positive effect) and this phenomenon differentiates the gain CRC groups from loss or CNLOH ones (Fig. 5A and C). Moreover, “Over-UpT CDIs” in selected chromosomes are significantly increased when compared to OverT (Fig. 5D) or Over-DownT CDIs (Fig. 5B), suggesting an enrichment of transcripts sensitive to BCNA-associated effect in the Over-UpT class. These results confirm that UpT are sensitive markers for BCNA-associated gene dosage effects, thus revealing a basic feature that distinguishes them from DownT.

In Fig. 5 the CDI values are not normalized for chromosomal transcript content and the CDI values of all T are reported for comparison. CDI can be normalized for the total number of transcripts synthesized in a chromosomal region (Normalized CDI, NCDI) according to the following formula:

$${\rm{NCDI}}\,{\rm{of}}\,{\rm{chromosomal}}\,{\rm{region}}\,n=\frac{{x}_{n}}{{X}_{n}}\,\ast \frac{1}{{\sum }_{i=1}^{T}\frac{{x}_{i}}{{X}_{i}}}\ast \,100$$

where *X*_*n*_ is the total number of transcripts transcribed in the *n-th* chromosomal region.

In Table 1 we report the normalized CDI (NCDI) of Over-UpT and Over-DownT synthesized in BGA-chromosomes or chromosomal arms. Data clearly show that NCDI of Over-UpT is the best parameter differentiating gain- from loss- and CNLOH-aberrant chromosomal regions. Table 1 also reports the number of Over-UpT and Over-DownT expressed as percentage of all T transcribed in the BGA-chromosomes (BGAT), in order to show that such parameter is not correlated with the type of chromosomal aberration.

**Table 1:** Over-UpT and Over-DownT expressed as CDI, NCDI, or percentage of transcripts synthesized in BGA regions (BGAT).

Selected CRC Group	Selected BGA-Chr	Over-UpT in BGA-Chr			Over-DownT in BGA-Chr		
		CDI	NCDI	% of BGAT	CDI	NCDI	% of BGAT
Chr20-gain	20	18.66	27.86	4.65	2.27	3.54	3.16
Chr8q-gain	8q	12.80	19.48	3.50	3.21	5.26	1.99
Chr13-gain	13	11.43	17.24	4.90	2.63	4.37	2.68
Chr7-gain	7	14.52	11.33	0.54	4.91	4.03	13.03
Chr9-gain	9	10.98	9.85	1.31	4.70	4.40	7.48
Chr18-loss	18	0.30	0.76	0.10	1.55	4.34	4.20
Chr8p-loss	8p	0.34	0.98	0.63	0.35	0.98	0.11
Chr6-CNLOH	6	5.82	3.74	1.25	8.50	5.95	1.27
Chr22-CNLOH	22	1.94	3.63	1.30	1.75	3.39	1.02

In order to evaluate if differences in RMA (robust multi-array average) transcript levels were the main determinants of the observed differences between Over-UpT and Over-DownT, we also report a comparison of the chromosomal distribution of Over-UpT with that of overexpressed transcripts that display RMA value >10 (a value approximately equal to the average +2 SD in CRC samples). The latter group of transcripts has been denominated as Over-HighT. As shown in Fig. 5D, although a significant enrichment of Over-HighT is observed in gain-aberrant Chr20, Chr8q and Chr13, but not in Chr7 and Chr9, the “Over-UpT CDI” in gain-aberrant chromosomes is significantly higher than “Over-HighT CDI” (Fig. 5D).

**Figure 6:**
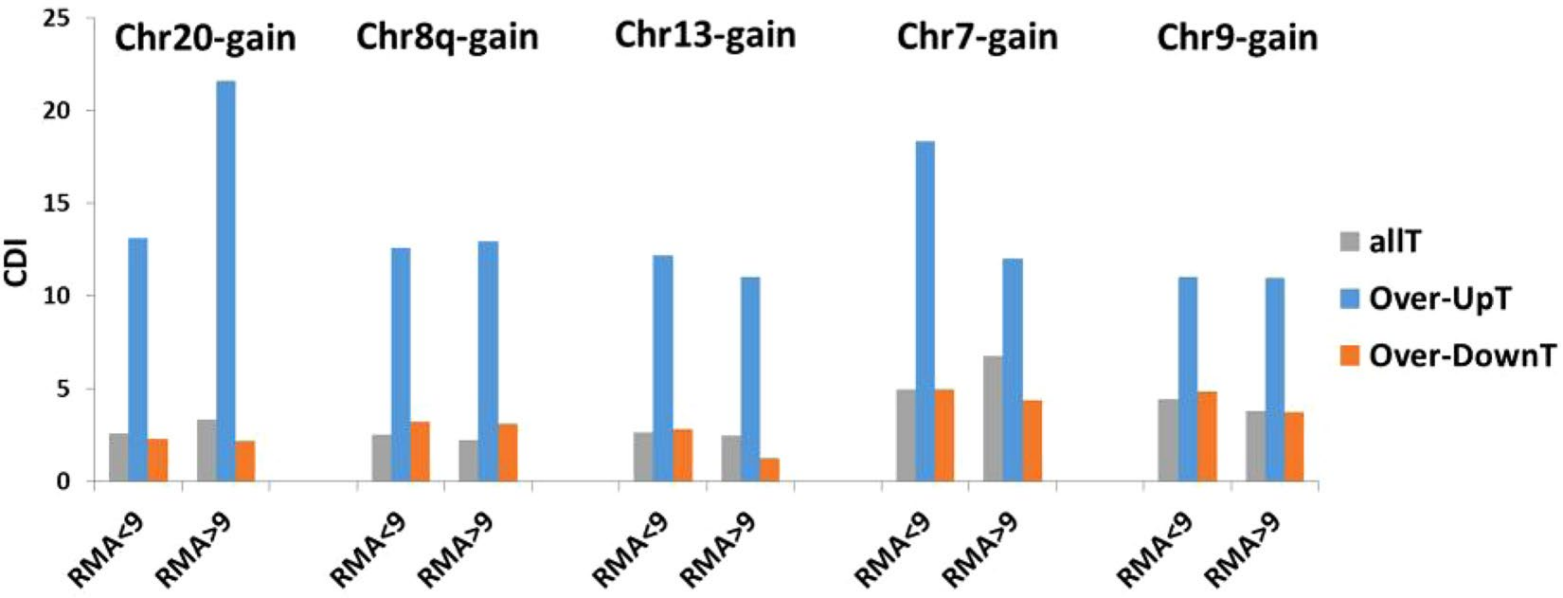
“Over-UpT” and “Over-DownT” classes. CDI of Over-UpT and Over-DownT in selected chromosomes of each Selected CRC group is reported as RMA averages. CDI of all transcripts analyzed in HTA (All T) is also reported for comparison. Transcripts were subdivided according to their expression level in two groups (low-expression transcripts with RMA <9 and high-expression transcripts with RMA >9).

A different analysis further confirmed that RMA transcript levels were not the main determinants of the observed differences between Over-UpT and Over-DownT. Transcripts were subdivided according to their expression level in two groups (low-expression transcripts with RMA <9 and high-expression transcripts with RMA >9) and analysis of CDI was performed as previously described. As shown in Fig. 6 the clear-cut difference between Over-UpT and Over-DownT CDI was confirmed in both groups of transcripts.

**Figure 7:**
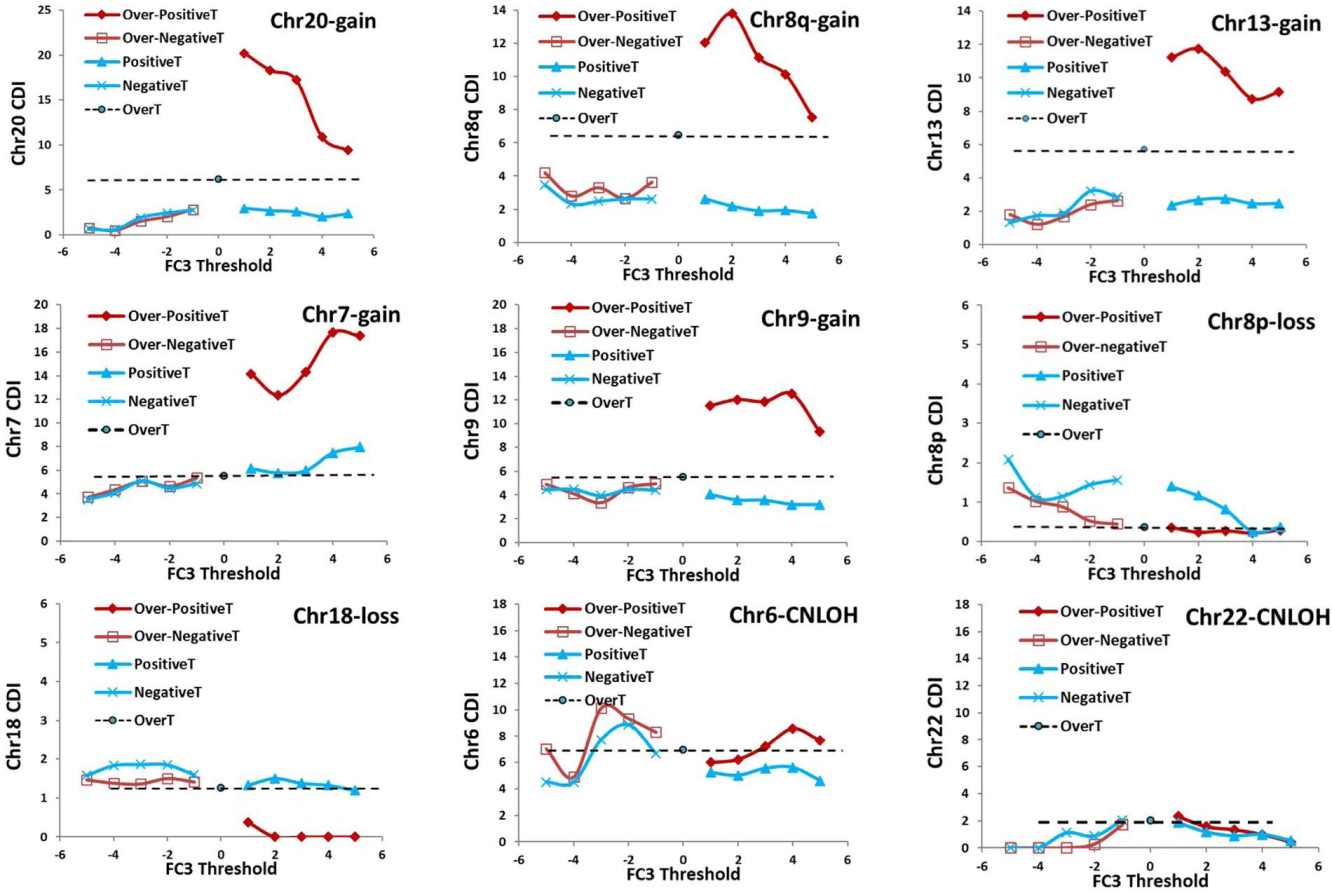
Varying FC3 thresholds for definition of transcript classes. CDI of Over-PositiveT, Over-NegativeT, PositiveT and NegativeT in selected chromosome of “gain”, “loss” and CNLOH CRC groups at different FC3 thresholds. CDI in selected chromosome for each group are calculated at progressively increasing FC3-threshold for PositiveT (transcripts with FC3 higher than threshold values shown in abscissa) and at progressively decreasing FC3-threshold for NegativeT (transcripts with FC3 lower than threshold values shown in abscissa). Horizontal dashed line indicates the CDI value of OverT.

Since transcript classes are based on the choice of arbitrary thresholds, we tested different values of FC3-threshold for the definition of classes. We called “Positive Transcripts” (PositiveT) all VT (FDR <0.05) showing positive values of FC3 and “Negative Transcripts” (NegativeT) those VT with negative values. It is clear that UpT of previous section are PositiveT above the fixed FC3-threshold of 1.5 and DownT are NegativeT under the fixed FC3-threshold of <−1.5. Figure 7 shows the plots obtained by progressively increasing the FC3-threshold for the selection of PositiveT and progressively decreasing it for the selection of NegativeT, while the FC2-threshold for OverT is maintained at a constant value (>1.3). An increased “Over-PositiveT CDI”, in comparison to “positive CDI”, can be observed in gain-aberrant chromosomes (Fig. 7) at all the cutoff values tested, while no significant increase is detected in loss-aberrant chromosomes and CNLOH groups (Fig. 7). In agreement with previous results no differences between “Over-NegativeT CDI” and “NegativeT CDI” are detected.

So far we analyzed the enrichment of Over-UpT in gain chromosomes by selecting UpT according to FC3 threshold. A similar analysis can be performed by selecting UpT genes according to FC4 threshold. As expected a comparison between Over-positiveT CDI calculated at varying FC3 or FC4 thresholds (Over-UpT CDI, corresponding to FC3 or FC4 >1.5, are also included in the plot) show only minor differences between the curves (Supplementary Fig. S2). In conclusion BCNA-associated cis-effect is mainly acting on positive transcripts (FC3 >1) and this effect can be denominated positive caricature transcriptomic effect.

We also modified the FC2 threshold while keeping constant the FC3 threshold at the established values for UpT (>1.5) or DownT (<−1.5). As shown in Supplementary Fig. S3 the FC2 cutoff value of 1.3–1.4 represents the best parameter to differentiate Over-UpT and OverT CDI. In agreement with previous results an increased CDI is observed in Over-UpT, but not in Over-DownT.

### List of Over-UpT and Under-UpT Genes and Cancer Gene Lists

**Figure 8:**
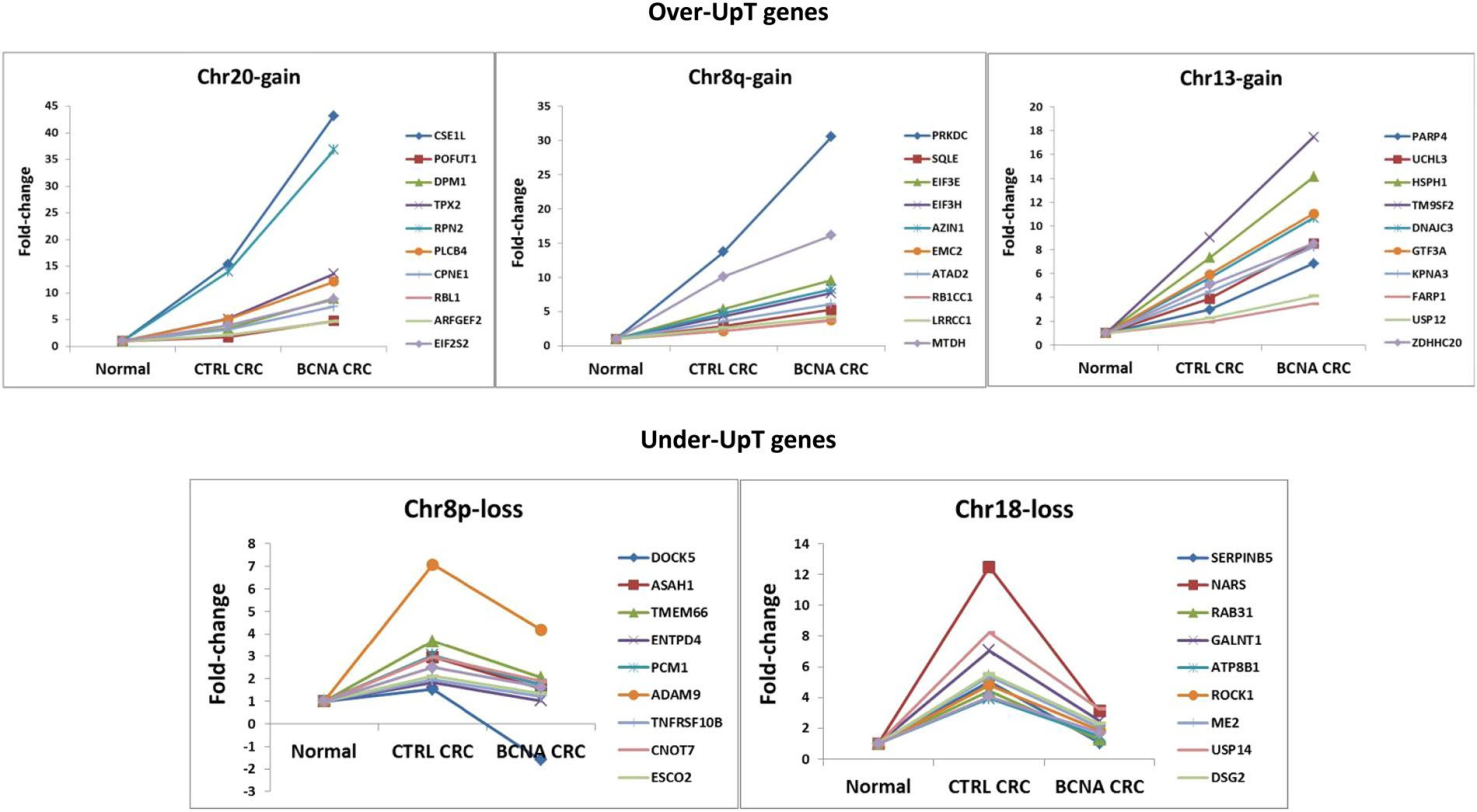
Fold changes of ten representative “Over-UpT” and “Under-UpT” genes. Over-UpT genes located in Chr20, Chr8q and Chr13 and Under-UpT genes located in Chr8p and Chr18. Ten representative genes for chromosome are shown. The plots show transcript levels in Normal Colonic Tissue (Normal), in Control (CTRL) CRC group and in selected CRC group (BCNA CRC). Transcript levels are expressed as fold-change relative to values in normal tissue (FC3 or FC4). A complete list of Over-UpT genes and their full names is reported in Supplementary Table S3. A complete list of Under-UpT genes and their full names is reported in Supplementary Table S4.

In order to generate a list of Over-PositiveT genes located in BGA regions we adopted the cutoff values FC2 >1.3 and FC3 >1.5, thus obtaining the list of the so-called Over-UpT genes. Fold-change values of ten representatives Over-UpT in Chr20, Chr8q and Chr13 are reported in Fig. 8. The complete list of Over-UpT genes in the selected CRC groups bearing gain-type BCNAs are reported in Supplementary Table S4.

In order to assess the presence of known cancer genes among Over-UpT genes we performed a comparison with three different lists: (1) 719 known cancer genes obtained from the Cancer Gene Census^41^, (2) 820 cancer-related genes, referred to as SCC-820, selected by Ohshima *et al*.^42^ in various databases and publications, (3) 299 cancer genes selected by Bailey *et al*.^43^ by merging results from 26 diverse bioinformatics tools and by manual curation.

In the Supplementary Table S5 we report the number of Over-UpT present in each selected chromosomes of the five gain groups (Chr20, Chr8q, Chr13, Chr7 and Chr9), the number of cancer driver genes of each list (CGC-719, SCC-820, CG-299) present on the same chromosomes and the number of cancer genes of each list belonging to the Over-UpT class; the names of such genes are reported in Supplementary Table S4. By hypergeometric test a significant enrichment of cancer genes among Over-UpT was observed in some chromosomes by using the three different lists (in Chr8q by CGC-719; in Chr20, Chr8q, Chr13 by SCC-820 and in Chr13 and Chr9 by CG-299). Moreover, when the five chromosomes (selected on the basis of the high frequency of gain-type BCNAs) are considered as a whole and the total number of genes are considered for statistical analysis, a significant enrichment was observed for each list (last column of Supplementary Table S5). It is relevant that a class of transcripts, selected for sensitivity to BCNA-induced transcriptional effects, shows a minor but significant enrichment of known cancer related genes. A larger enrichment was not expected since Over-UpT have been selected for their association to BCNAs (mild increase of copy number <4), while the majority of known cancer genes are selected for their association to point mutations, gene fusions and high-level (>4) amplifications.

When Over-UpT genes are ranked according to their overexpression levels, several genes already implicated at the protein, functional and therapeutic level in cancer, can be observed in the top positions, such as CSE1L in Chr20^44,45^, PRKDC, encoding DNA-dependent protein kinase catalytic subunit^46^, and SQLE (squalene epoxidase)^47^ in Chr8q, HSPH1 (encoding HSP110)^48^ and CDK8^39,49^, in Chr13, MACC1^50^ in Chr7, CKS2 (Cyclin-dependent kinases regulatory subunit 2^51,52^, and SET oncogene^53^ in Chr9.

Six of the top ten Over-UpT genes in Chr20 have been involved in tumorigenesis based on overexpression, functional assays and clinical data: CSE1L^44,45^, POFUT1^54^, TPX2^55^ RPN2^56^, PLCB4^57^, CPNE1^58,59^.

Since the products of BCNA-associated cancer driver genes are predicted to act in cooperative manner, the lists of Over-UpT genes were also examined for the presence of strictly functionally cooperating proteins, such as subunits of protein complexes. Two genes encoding for eukaryotic translation initiation factors (EIF3E and EIF3H) are located in Chr8q. Their transcript levels are 4–5 fold higher in cancer samples with a disomic Chr8 in comparison to normal tissue and overexpressed comparing cancer samples bearing Chr8q gain with those with disomic Chr8 (1.8-fold change). Interestingly, as reported in Supplementary Table S4, another two genes encoding for eukaryotic translation initiation factors (EIF2S2 and EIF6) are located in Chr20 and are significantly upregulated in comparison to normal tissue (4- and 2-fold change, respectively) and overexpressed comparing cancers bearing Chr20 gain with those with disomic Chr20 (2.2 and 1.67-fold change, respectively). Among Over-UpT genes located in Chr8q we also report the presence of MTDH (metadherin) and AGO2, whose products are members of the RNA-induced silencing complex (RISC).

Regarding the strong anticaricature effects on UpT (increased “Under-UpT CDI”) observed in loss-aberrant chromosomes, a list of Under-UpT genes is reported in Supplementary Table S6 and a plot of fold-change values of ten representative transcripts per chromosome is shown in Fig. 8. As an example of a Chr8p-located gene, the TNFRSF10B (tumor necrosis factor receptor superfamily, member 10b, also known as Death Receptor 5) is upregulated (1.95-fold increase) in CRC samples in comparison to normal tissue but its expression decreases in CRC samples bearing a Crh8p loss. Other putative TSGs, such as SMAD4, are present among Under-UpT genes located in Chr18.

### RNA-Seq Transcriptome Profiling Data from TCGA-COAD Project

**Figure 9:**
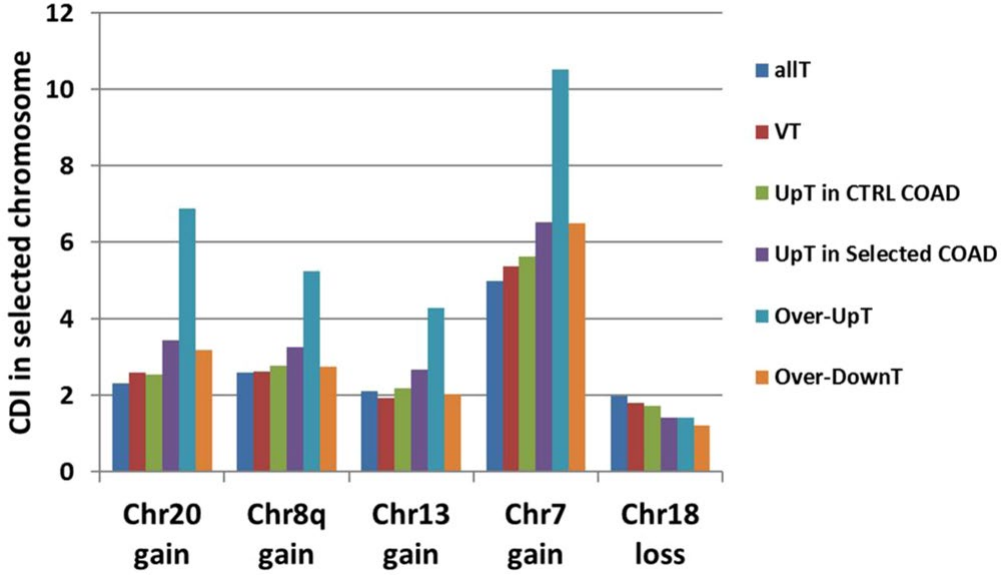
“Up-T”, “Over-UpT” and “Over-DownT” classes in the TCGA-COAD samples. CDI of Up-T, Over-UpT and Over-DownT has been calculeted in selected chromosomes of each Selected CRC group using TPM values obtained by RNA-seq in TCGA-COAD samples. CDI of all transcripts analyzed in RNA-seq (All T) is also reported for comparison. *p < 0.01 comparing the selected chromosome versus all other chromosomes by chi-squared test.

In order to compare results obtained in our cohort of CRC samples, we downloaded RNA-Seq transcriptome profiling data and masked copy number segment files (obtained by Affymetrix SNP 6.0 array) relative to the COAD (Colon adenocarcinoma) project (The Cancer Genome Atlas, TCGA) from GDC Data Portal (NIH, National Cancer Institute, https://portal.gdc.cancer.gov/). Both raw counts and FPKM data (converted in TPM according to Wagner *et al*.^60^) were downloaded for RNA-Seq analysis. Since the complete dataset of COAD contained only 41 normal colonic mucosae, we selected the 46 tumor samples deriving from the same patients so to have a group of matched tumors and normal tissues. On the basis of copy number data, we formed 5 selected CRC groups (Chr20-gain, Chr8q-gain, Chr13-gain, Chr7-gain, Chr18-loss) and corresponding control CRC groups (number of samples for each group are reported in Supplementary Table S7). As shown in Fig. 9 data confirmed the increase of Over-UpT CDI in gain CRC groups and the lack of sensitivity of Over-DownT to BCNA-associated gene dosage effect. A large percentage of Over-UpT genes in the list compiled by microarray data were confirmed by the RNA-Seq data (66% in Chr20, 36% in Chr8q, 39% in Chr13, 57% in Chr7; names of the Over-UpT genes confirmed by RNA-Seq in COAD samples are indicated in Supplementary Table S3).

## Discussion

In the present paper we report an integrative analysis of gene copy number and transcriptomic data in colorectal cancer with a focus on BCNAs, defined as whole-chromosome or arm-level copy number aberrations. Several previous works have been dedicated to similar integrative analysis, but the majority have concentrated their efforts on focal and high-level gene amplifications^7,8,33,36,37^.

Recently, Ohshima *et al*.^42^ reported an integrative analysis of copy number alterations and gene expression, suggesting that overexpression (corresponding to upregulation in our terminology) is a requisite for amplified genes to function as driver alterations. As an index of amplification-dependent overexpression of a specific gene they use the percentage of co-occurrence of amplification and overexpression out of the total cases of overexpression of that gene. However, the simple association of overexpression (higher expression in cancer in comparison to normal tissues) and moderate gene amplification (copy number >3 and <5 such as those observed in gain-type BCNAs) does not necessarily imply a causal relationship between amplification and upregulation (i.e. amplification-dependent upregulation). Moreover, they restrict their analysis to lists of known cancer genes and their study is focused on determining which genes, in a list of already known or strongly suspected cancer genes, can be activated by the mechanism of amplification-dependent overexpression.

In our study we used an alternative approach: we evaluated the possible influence of copy number gains on gene expression by comparing tumor groups differing for the presence of a specific gain-type BCNA. Transcripts showing a significant higher expression in BCNA-bearing tumors in comparison to control tumors are indicated as overexpressed (OverT). However, the list of OverT genes only partially reflects the BCNA-sensitive genes for at least two reasons: (1) inclusion in OverT classes depends on the choice of a threshold; (2) several other cancer-associated transcriptional dysregulation processes, due to mutational and epimutational differences, differentiate the two CRC groups and contribute to the appearance of OverT and UnderT^61^. Indeed, Fehrmann *et al*.^34^ reported that only 28% of the total expression variation between genomically stable and unstable human samples was strongly correlated with copy number variation and differences in expression profiles between class of tumors with different form of genomic instability are well known^39^. In order to provide an estimate of the association between OverT and the specific gain-type BCNA we used the variations of an index called the chromosomal distribution index (CDI). The assumption behind the adoption of such quantitative index is that prior knowledge on the physical localization of genes can be useful in roughly estimating the proportion of OverT associated to BCNA-dosage effects. Indeed, one of the result of our work is that CDI shows significant variations in aberrant chromosomes selected for the formation of the cancer groups in a direction concordant with the type of BCNA (gain or loss). The presence of major CDI changes in selected aberrant chromosomes was true in different groups selected for gain- or loss-BCNAs and did not occur in two tumor groups formed on the basis of copy-neutral aberrations (CNLOH). On the basis of these results we used quantitative changes of CDI in selected chromosomes (aberrant chromosomes used for tumor group formation) as an index of sensitivity of different class of transcripts to BCNA-dosage effects. The largest variations of CDI in selected gain chromosomes were observed for the Over-UpT class (transcripts that are overexpressed in BCNA-bearing cancer in comparison to control cancer and upregulated in control cancer in comparison to normal colonic tissue), while no significant variations were observed for Over-DownT classes (transcripts that are overexpressed in BCNA-bearing cancer in comparison to control cancer but downregulated in control cancer in comparison to normal tissue). Therefore, another result of the study is that cancer up-regulated genes are enriched among genes sensitive to BCNA-associated gene dosage effects. We called this effect “BCNA-associated positive caricature transcriptomic effects”, based on the definition of “caricature” as an exaggeration of a tumor’s distinctive features.

This difference is not linked to the technology used for transcriptome analysis, as shown by the fact that we could reproduce similar results by using the transcriptome data generated through RNA-Sequencing (RNA-Seq) by the TGCA-COAD project. As far as global transcriptomic effects are concerned data by RNA-Seq are in agreement with microarray results.

Fehrmann *et al*.^34^ have reported that genes abundantly expressed were more dosage sensitive than genes with low expression levels. Our data confirm that genes within a high range of expression level (RMA values >10) show an increased CDI in BCNA-chromosomes (Over-HighT genes). However, this effect is detectable only in a fraction of BCNA chromosomes (Chr20, Chr8q and Chr13) and is significantly lower than that observed with Over-UpT. Moreover, when transcripts are divided in highly expressed and moderately expressed the difference in dosage sensitivity between Over-UpT and Over-DownT is maintained (Fig. 6).

Previous results do not give any information on the presence of cancer driver genes (number or identity) among Over-UpT genes located in BCNAs. An estimate of the minimal value of fold-enrichment of cancer genes among BCNA-located Over-UpT genes has been obtained by comparisons with various lists of known cancer driver genes, yielding a low, but significant, value of 3.45–5.45 fold-enrichment in comparison to expectations. The low value is explained by the fact that most known cancer genes have been selected on the basis of point mutations, gene fusions or high level amplifications and such criteria do not correspond to the features of putative BCNA-associated cancer driver genes.

We are not suggesting that Over-UpT are cancer driver genes, but that this class of transcripts can be prioritized for further functional studies on such specific type of cancer genes (BCNA-associated). Indeed, the list of Over-UpT genes in Chr20, ranked according to overexpression, shows in the top positions several bona fide cancer driver genes, already implicated in cancer pathogenesis on the basis of evidence at the protein, functional and clinical level^44,45,54–59,62^. Moreover, it should be kept in mind that transcript level changes are not necessarily translated in phenotypic effects and several downstream mechanisms can counterbalance such modifications^63,64^.

In the list of Over-upT genes in Chr8q we also noted the presence of subunits of protein complexes, such as the eukaryotic translation initiation factors eIF3E and eIF3H. Indeed, it has been reported that increased expression of EIF3H gene increases CRC growth and invasiveness^65^. Moreover, Gandin *et al*.^66^ have reported that another eukaryotic translation initiation factors, eIF6, included in the list of Over-UpT genes located in Chr20, is rate-limiting in translation, growth and transformation. Another example of functional interaction between proteins encoded by Over-UpT genes in Chr8q is represented by MTDH and AGO2, whose products are members of the RNA-induced silencing complex (RISC). MTDH (also known as AEG1) has been considered as an oncogene in melanoma, malignant glioma, breast cancer and hepatocellular carcinoma^67^. Of course these are only examples of functional interactions involving the products of Over-UpT genes. Their possible roles, as rate-limiting steps in the functional activity of pathways linked to cancer progression, need to be further investigated.

In agreement with the specificity of the phenomenon for gain-type BCNAs no caricature effects on UpT are observed in chromosomes bearing loss-type BCNA or CNLOH. On the contrary, the most evident effect associated with loss-type BCNAs is an “anticaricature effect” on UpT genes located in Chr8p (i.e. several UpT genes are underexpressed in the presence of Chr8p-loss). It is known the transcription of some TSGs can increases during cancerogenesis as an extreme compensatory mechanism that opposes to cancer growth. Loss of a chromosomal arm may be responsible for their decreased transcriptional activity, thus facilitating cancer progression. As an example of an Under-UpT gene with potential tumor-suppressor activity we report the TNFRSF10B gene (also known as DR5). This gene encodes for one of the receptors for TRAIL, a potent death-inducing ligand that mediates apoptosis and serves as an important endogenous tumor suppressor mechanism^68^. Another example is provided by SMAD4, a TSG involved in the signal transduction cascade initiated by TGF-β superfamily and in CRC pathogenesis. SMAD4 is located in Chr18, the most frequently deleted chromosome in CRC, and shows the Under-UpT transcriptional profile typical of the anticaricature effect. This result is in agreement with previous data showing an increased expression of SMAD4 mRNA in colon adenomas and a decreasing expression with tumor progression^69^. We are not proposing that most genes with an Under-UpT transcriptional profile are classical TSGs (Type I TSG). Indeed, the majority of genes present in such category would not fit the actual experimental criteria for TSGs. Under-UpT could be candidate as cancer driver genes involved in cumulative haploinsufficiency, but different approaches are required for experimental validation.

We have observed only minor transcriptional effects in genes located in CNLOH-bearing chromosomes, such as a decreased anticaricature effect on UpT. This weak effect is in agreement with the presence of a normal gene copy number in this type of chromosomal aberration and could reflect the existence of subtler changes in gene transcription. Indeed, it has been proposed that expression of selected genes included in broad CNLOH region can be modified by gene dosage independent mechanisms^17^ and it is conceivable an impact of those expression changes on cancer phenotype. For instance, CNLOH in imprinted genomic regions may produce loss-of-imprinting or gain-of imprinting of selected genes and cumulative changes in gene expression^25^.

In conclusion, our results show that cancer up-regulated genes are enriched among genes sensitive to BCNA-associated gene dosage effects. To our knowledge this is the first description of the BCNA-associated positive caricature transcriptomic effect and we suggest that this effect could be exploited to prioritize candidate BCNA-associated cancer genes for experimental validation.

## Methods

### Genome-Wide DNA Copy Number and SNP Genotyping Analysis in CRC samples

Genome-wide DNA copy number and SNP genotyping analysis was performed on Affymetrix SNP 6.0 arrays (Affymetrix, Inc., Santa Clara, CA, USA), using 500 ng of input DNA. Tumor samples were collected from a cohort of 50 patients who underwent resection of primary CRC at “Centro Clinico Diagnostico S.r.l. G.B. Morgagni” in Catania (Italy). The study was approved by the Ethics Committee of ASL3 of Catania (Italy) and all methods were performed in accordance with relevant guidelines and regulations for research involving human participants. All patients gave informed consent for the study. Specimens were frozen and stored at −80 °C until DNA extraction. In 29 cases biopsies of adjacent normal mucosa (at distance of 3–6 cm from the tumor) were also collected (matched tumor/mucosa pairs). For 16 patients, two biopsies were taken from the same tumor mass, at a distance ≥1 cm from each other (double-sampling pair). In one case, in addition to a double sampling, a synchronous tumor located in another site of the colon was present and biopsied.

Array scanning and data analysis were performed by using the Affymetrix® “GeneChip Command Console” (AGCC) and the “Genotyping Console™” (GTC) version 3.0.1 software. Broad Copy Number Abnormalities (BCNAs), defined as gains or losses involving more than 25% of a chromosomal arm or numerical aberrations involving whole chromosomes, were identified by using a bioinformatic tool called BroCyA, as described by Barresi *et al*.^38^.

Raw and processed data of SNP 6.0-array results have been submitted to public repository: “Gene Expression Omnibus-GEO” (www.ncbi.nlm.nih.gov/geo) with the following accession number: GSE80460.

### Microsatellite Testing, Consensus Molecular Subtype Classifier and KRAS Mutation Detection

Samples were tested for microsatellite instability with five markers belonging to the Bethesda panel (D2S123, D5S346, D17S250, BAT25 and BAT26) and one additional marker (BAT40). Tumors were defined as MSI if ≥30% markers were found unstable when comparing tumor versus normal colonic tissue. Consensus molecular subtypes (CMS) of colorectal cancer were determined according to Guinney *et al*. 2015 by an R package (CMSclassifier), which included the Single Sample Predictor (SSP) classifier. For KRAS Sanger sequencing, codons 12 and 13 of KRAS exon 1 were amplified and PCR products purified and sequenced on an ABI Prism 310 Genetic Analyzer (TermoFisher Scientific, MA, USA) with the Applied Biosystems BigDye terminator cycle sequencing ready reaction kit (TermoFisher Scientific, MA, USA).

### Transcriptome Analysis

Transcriptome analysis (67528 transcripts) was performed from 100 ng of total RNA by amplification and target hybridization to the Gene-Chip Human Transcriptome Array (HTA) 2.0 (Cat. No. 902310, Cat. No. 900720; Affymetrix, Inc., Santa Clara, CA, USA). Array scanning and data analysis were performed with the Affymetrix® Expression Console^TM^ software version 1.4 (Affymetrix, Inc., Santa Clara, CA, USA) and the Affymetrix® Transcriptome Analysis Console (TAC) software (Affymetrix, Inc., Santa Clara, CA, USA). Transcript level analysis was performed using the normalization method based on the processing algorithm called robust multi-array average (RMA)^70^. Linear fold-changes were calculated in the following way: 2^[study group Average RMA – control group average RMA]^ if study group > control group, or −2^[control group Average RMA – study group average RMA]^ if study group < control group. 46 tumoral samples and 26 normal matched mucosae were analyzed and data have been deposited to public repository “Gene Expression Omnibus-GEO” (www.ncbi.nlm.nih.gov/geo) and will be accessible through GEO: GSE73360^71^ and GSE84984.

### Changes in Chromosomal Distribution Index

The statistical significance of the chromosomal enrichment or depletion of a transcript class in a BGA-region (changes in CDI) was calculated by chi-squared test. Data of Figs 3, 4E and 5A–C were analyzed by 2 * 2 contingency table containing number of genes belonging or not belonging to a transcriptional class located in a specific chromosome (or chromosomal arm) or in all other chromosomes (or chromosomal arms). Data of Fig. 4A,C were analyzed by 2 * 2 contingency tables containing number of genes belonging to a transcriptional class located in a specific chromosome (or chromosomal arm) or in all other chromosomes (or chromosomal arms) in selected or control CRC groups. Data in Fig. 5D were analyzed by 2 * 2 contingency table containing number of Over-UpT genes or OverT (or Over-HighT) located in a specific chromosome or in all other chromosomes.

### Analysis of cancer gene lists

The probability (p-value) that Over-UpT genes are overlapping no less than “x” cancer genes in three different lists (719 genes downloaded on June 2018 at http://cancer.sanger.ac.uk/census/41; 820 cancer-related genes, referred to as SCC-820^42^; 299 cancer genes^43^ by random chance was calculated according to a hypergeometric distribution model according the following equation:

$${\rm{P}}=1-{\sum }_{k=0}^{x-1}\frac{(\begin{array}{c}r\\ k\end{array})(\begin{array}{c}N-r\\ n-k\end{array})}{(\begin{array}{c}N\\ n\end{array})}$$

where “N” is the total number of genes located in the selected whole chromosome or chromosome arm, “r” is the number of cancer genes located in the selected whole chromosome or chromosome arm, “n” is the number of Over-UpT genes located in the selected chromosome, and “x” is the number of cancer genes, located in the selected whole chromosome or chromosome arm, that are overexpressed and upregulated.

### Ethics, consent and permissions

Tumor samples were collected from patients who underwent surgical resection at “Centro Clinico Diagnostico S.r.l. G.B. Morgagni” in Catania (Italy). The study was approved by the Ethics Committee of ASL3 of Catania (Italy) and all methods were performed in accordance with relevant guidelines and regulations for research involving human participants. All patients gave informed consent for the study.

## Electronic supplementary material

Supplementary Figures and Tables

## Data Availability

All data generated or analyzed during this study are included in this published article (and its supplementary files) and in public repository: “Gene Expression Omnibus-GEO” (www.ncbi.nlm.nih.gov/geo) with the following accession number: GSE80460; GSE73360 and GSE84984.
