# Supplementary material for: Positive Caricature Transcriptomic Effects Associated with Broad Genomic Aberrations in Colorectal Cancer
Source: Sci Rep. 2018 Oct 4;8:14826. doi: 10.1038/s41598-018-32884-3 (PMC6172234; doi:10.1038/s41598-018-32884-3)
Supplement: Supplementary file 1 — Supplementary Figures and Tables [file 41598_2018_32884_MOESM1_ESM.pdf]

Positive Caricature Transcriptomic Effects Associated with Broad Genomic Aberrations in Colorectal Cancer.

Daniele F. Condorelli[1*], Giorgia Spampinato[1], Giovanna Valenti[1], Nicolò Musso[1], Sergio Castorina[2], Vincenza Barresi[1*]

[1]Department of Biomedical and Biotechnological Sciences, Section of Medical Biochemistry, University of Catania, Catania (95123) - Italy

[2]Department of Medical and Surgical Sciences and Advanced Technologies, University of Catania, Catania (95123) - Italy

*Corresponding Authors:

Prof. Daniele F. Condorelli, MD, PhD (daniele.condorelli@unict.it)

Prof. Vincenza Barresi, PhD (barregi@unict.it)

Section of Medical Biochemistry, Department of Biomedical and Biotechnological Sciences

University of Catania, Catania

Via S. Sofia 89-97, 95123 CATANIA - Italy

Supplementary Figure S1. CDI (% of transcripts) of OverT and UnderT in all chromosomes of each selected CRC group.

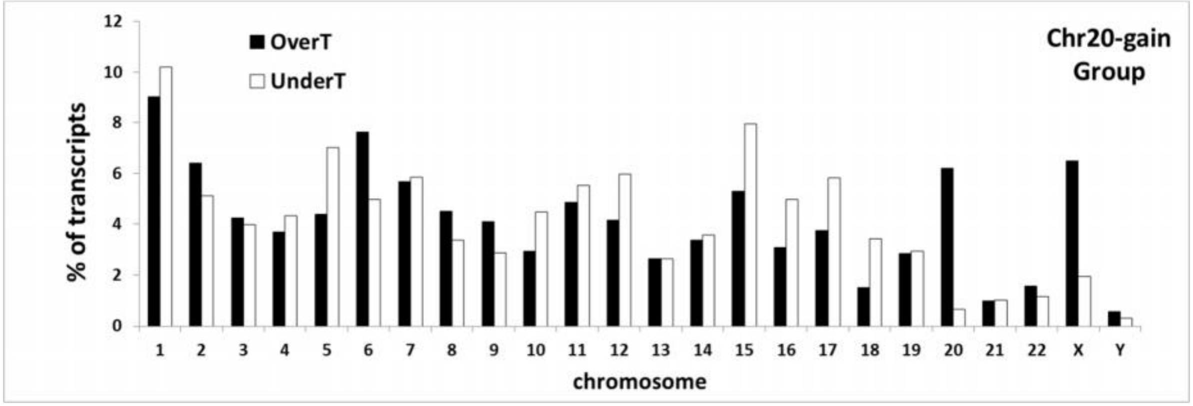

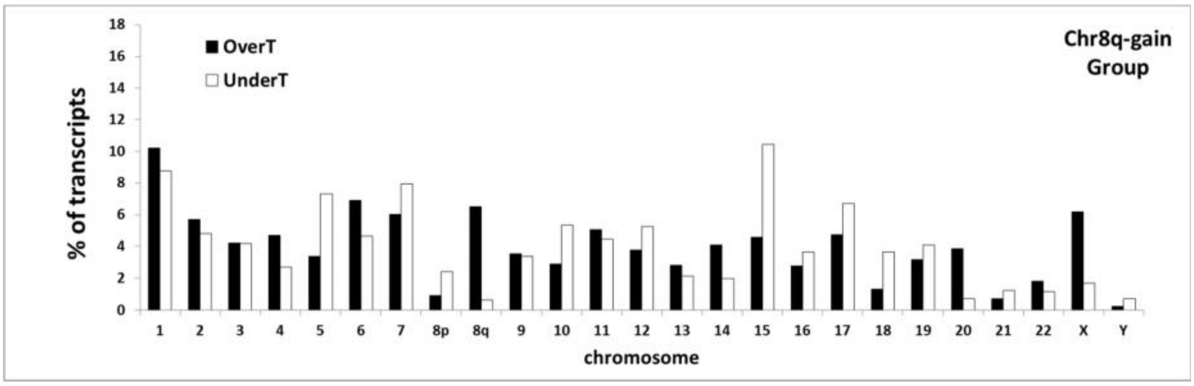

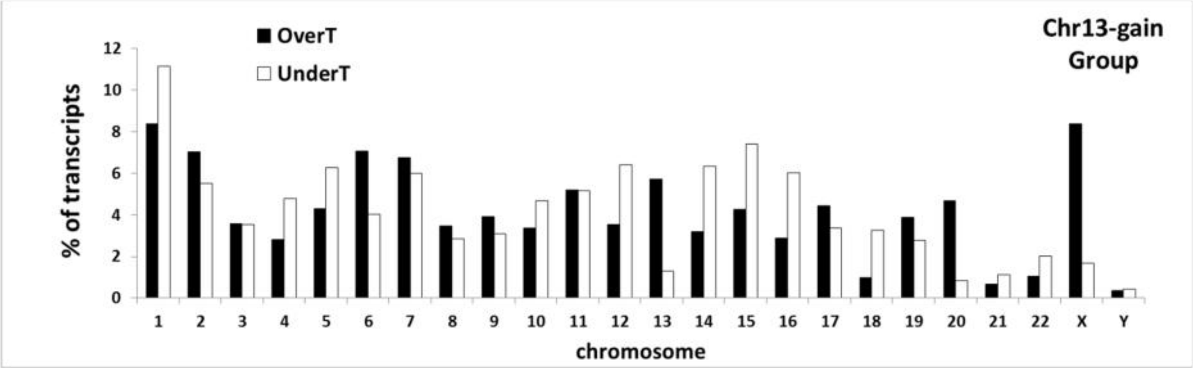

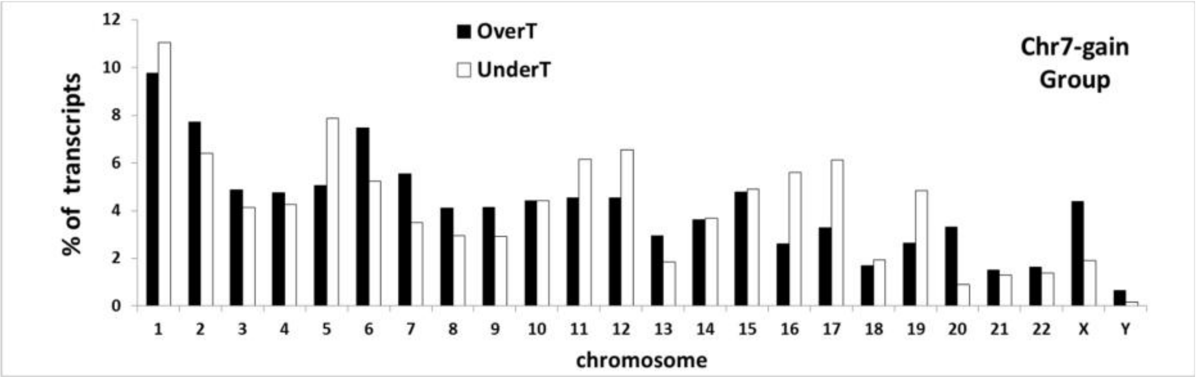

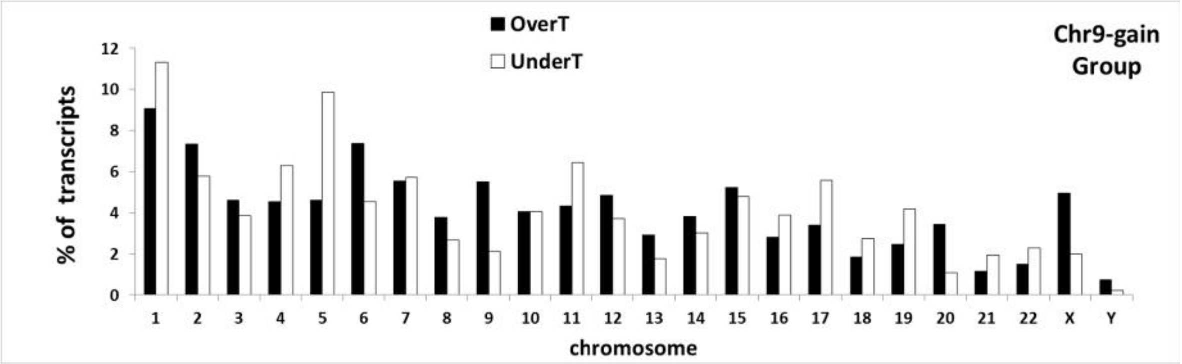
OverT
UnderT
Chr9-gain
Group
12
10
8
6
4
2
0
% of transcripts
1 2 3 4 5 6 7 8 9 10 11 12 13 14 15 16 17 18 19 20 21 22 X Y
chromosome

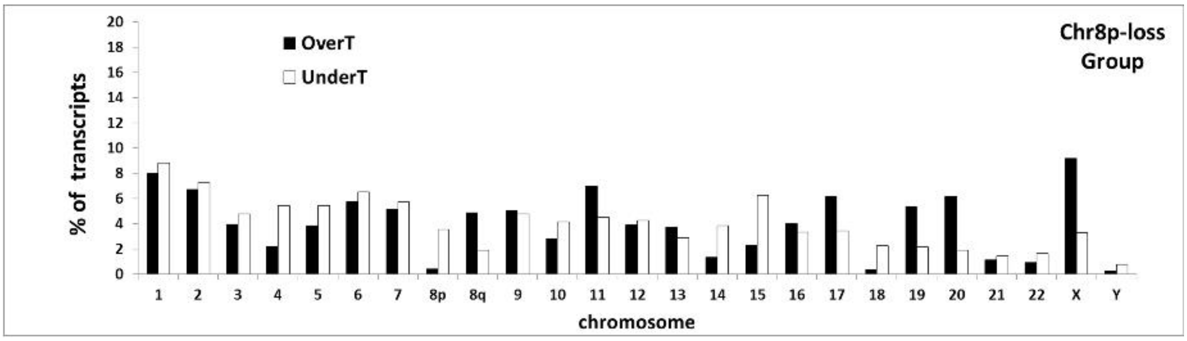
OverT
UnderT
Chr8p-loss
Group
20
18
16
14
12
10
8
6
4
2
0
% of transcripts
1 2 3 4 5 6 7 8p 8q 9 10 11 12 13 14 15 16 17 18 19 20 21 22 X Y
chromosome

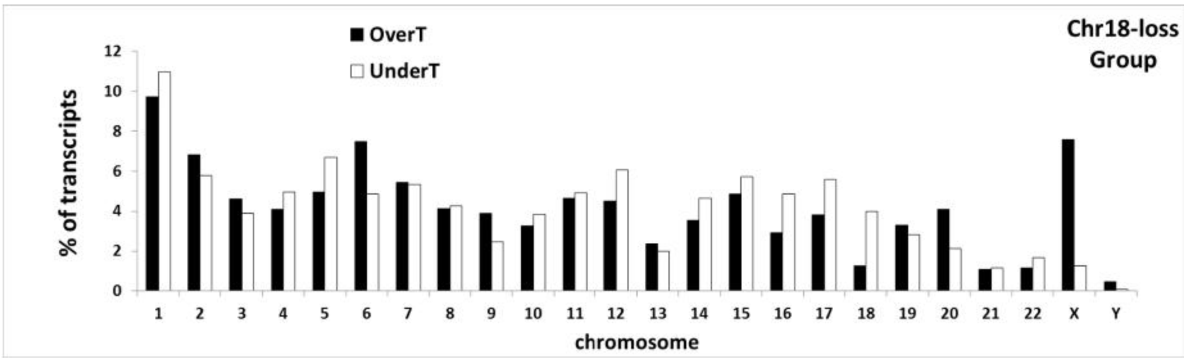
OverT
UnderT
Chr18-loss
Group
12
10
8
6
4
2
0
% of transcripts
1 2 3 4 5 6 7 8 9 10 11 12 13 14 15 16 17 18 19 20 21 22 X Y
chromosome

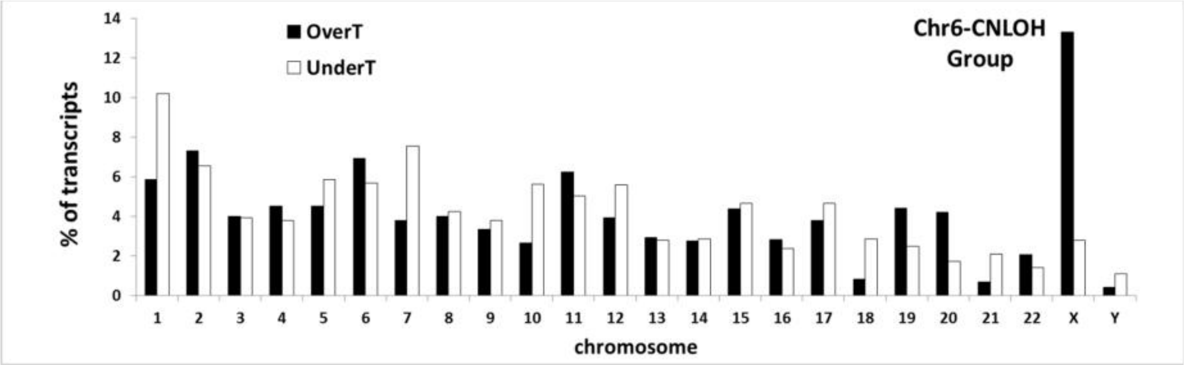
OverT
UnderT
Chr6-CNLOH Group
% of transcripts
chromosome
1 2 3 4 5 6 7 8 9 10 11 12 13 14 15 16 17 18 19 20 21 22 X Y
0 2 4 6 8 10 12 14

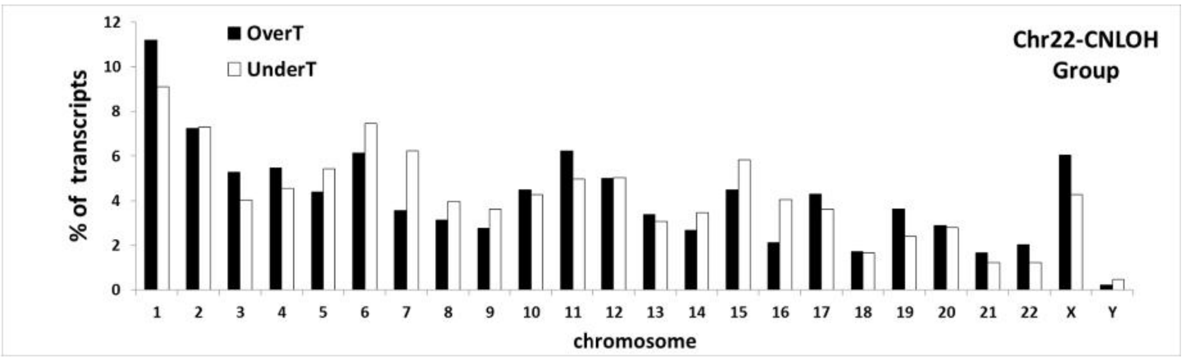
OverT
UnderT
Chr22-CNLOH Group
% of transcripts
chromosome
1 2 3 4 5 6 7 8 9 10 11 12 13 14 15 16 17 18 19 20 21 22 X Y
0 2 4 6 8 10 12

Supplementary Figure S2. CDI of Over-PositiveT in selected chromosome of gain CRC groups at different FC3 or FC4 thresholds. CDI in selected chromosome for each group are calculated at progressively increasing FC3- or FC4-threshold (higher than the value indicated in abscissa) while keeping constant the FC2 threshold (>1.3). Horizontal dashed line indicates the CDI value of OverT (FC2>1.3; no threshold for FC3 and FC4).

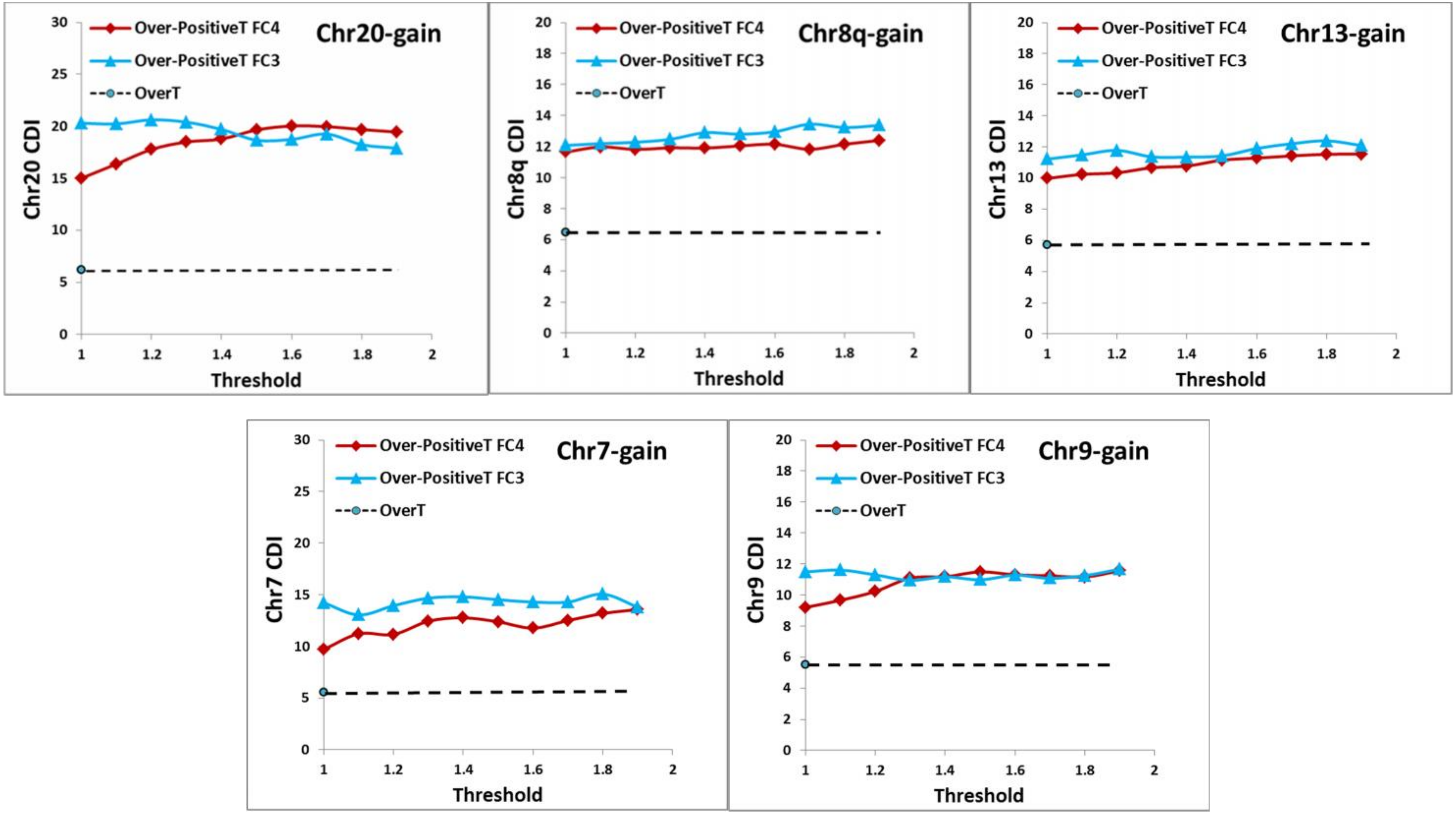

Supplementary Figure S3. Varying FC2 thresholds for definition of transcript classes**.** CDI of Over-UpT and Over-DownT in selected chromosome of gain CRC groups at different FC2 thresholds. CDI in selected chromosome for each group are calculated at progressively increasing FC2-threshold (higher than the value indicated in abscissa) while keeping constant the FC3 threshold for UpT (>1.5) or DownT (<-1.5).

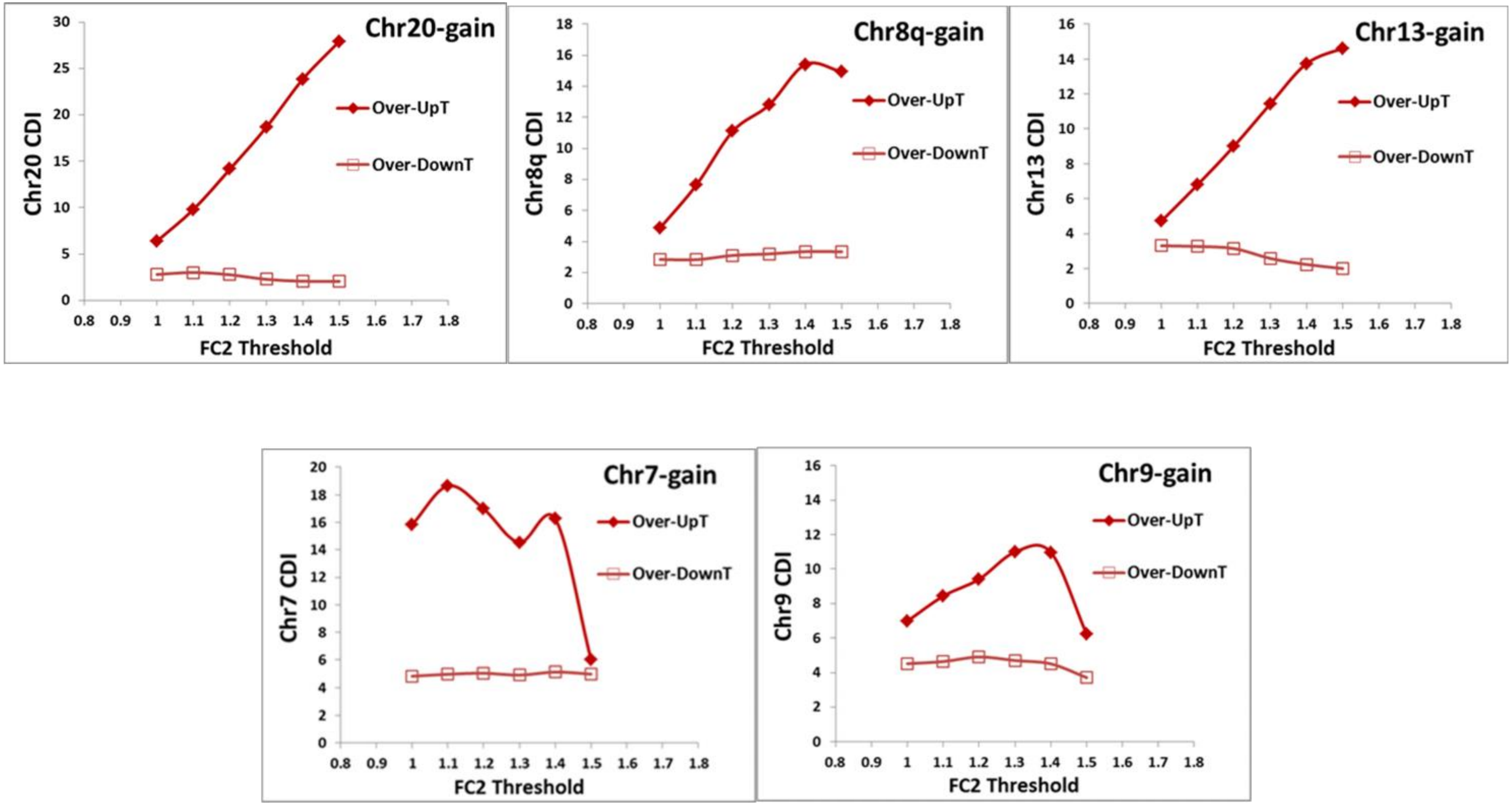

Supplementary Table S1. Clinicopathological (age, sex, anatomical site, stage) and molecular (microsatellite instability status, activating KRAS mutations, Consensus Molecular Subtypes according to Guinney et al., 2015) features are reported and as expressed as percentage of samples*. According to the American Joint Committee on Cancer (AJCC) staging system§.

Genomic aberration	Chromosome group	Sample group	N. samples	Age at surgery (mean ± SD)	Gender (% of male)	Anatomical site*			Tumor stage (AJCC)§;*			MSI status*	KRAS mutation*	Consensus Molecular Subtype classification*				
						right	left	rectum	2	3	4			CMS1	CMS2	CMS3	CMS4	NA
GAIN	Chr20-gain	Control CRC	19	69.1 ± 14.5	53.8	68.4	15.8	15.8	36.8	57.9	5.3	26.3	52.6	26.3	26.3	15.8	5.3	26.3
		Selected CRC	27	67.5 ± 16.1	60.0	40.7	48.2	11.1	44.4	44.4	11.2	0.0	29.6	0.0	81.5	7.4	3.7	7.4
	Chr8q-gain	Control CRC	17	67.5 ± 13.8	69.2	47.1	35.3	17.6	29.4	64.7	5.9	11.8	41.2	17.6	52.9	17.6	0.0	11.8
		Selected CRC	28	70.5 ± 14.2	50.0	53.6	35.7	10.7	53.6	39.3	7.1	10.7	35.7	7.1	75.0	3.6	7.1	7.1
	Chr13-gain	Control CRC	16	68.5 ± 14.2	46.2	50.0	18.8	31.2	43.8	50.0	6.2	18.8	43.8	18.8	50.0	18.8	0.0	12.5
		Selected CRC	27	70.7 ± 13.6	52.4	48.1	48.1	3.8	44.4	48.2	7.4	7.4	33.3	7.4	74.1	0.0	7.4	11.1
	Chr7-gain	Control CRC	19	70.3 ± 13.9	60.0	31.6	52.6	15.8	42.1	52.6	5.3	21.1	42.1	26.3	36.8	15.8	5.3	15.8
		Selected CRC	22	68.8 ± 15.6	60.0	50.0	36.4	13.6	36.4	50.0	13.6	4.5	31.8	0.0	90.9	4.5	0.0	4.5
	Chr9-gain	Control CRC	29	71 ± 12,33	57.1	69.0	17.2	13.8	41.4	51.7	6.9	17.2	34.5	17.2	55.2	13.8	3.4	10.3
		Selected CRC	9	64.9 ± 21.9	50.0	44.4	55.6	0.0	44.5	33.3	22.2	0.0	55.6	0.0	77.8	0.0	11.1	11.1
LOSS	Chr18-loss	Control CRC	18	75.9 ± 11.7	57.1	72.2	11.1	16.7	44.4	55.6	0.0	22.2	38.9	27.8	38.9	11.1	5.6	16.7
		Selected CRC	26	64.8 ± 15.4	55.0	42.2	46.3	11.5	46.2	42.3	11.5	3.8	42.3	0.0	80.8	7.7	3.8	7.7
	Chr8P-loss	Control CRC	23	73.4 ± 12.6	55.0	65.2	17.4	17.4	47.8	47.8	4.4	21.7	43.5	21.7	43.5	17.4	4.3	13.0
		Selected CRC	19	65.9 ± 15.2	53.8	36.8	57.9	5.3	42.1	47.4	10.5	0.0	36.8	0.0	89.5	0.0	5.3	5.3
CNLOH	Chr6-CNLOH	Control CRC	31	71.4 ± 12	64.0	64.5	29.0	6.5	35.5	54.8	9.7	16.1	38.7	9.7	64.5	12.9	3.2	9.7
		Selected CRC	8	61.6 ± 17	0.0	37.5	37.5	25.0	25.0	75.0	0.0	0.0	50.0	0.0	62.5	0.0	12.5	25.0
	Chr22-CNLOH	Control CRC	32	67.6 ± 16.8	62.5	62.5	25.0	12.5	46.9	43.8	9.3	9.4	37.5	12.5	59.4	9.4	6.3	12.5
		Selected CRC	6	68.7 ± 15.3	0.0	66.7	33.3	0.0	33.3	66.7	0.0	0.0	33.3	16.7	66.7	16.7	0.0	0.0

*Each value as expressed as percentage of samples

§According to the American Joint Committee on Cancer (AJCC) staging system.

Supplementary Table S2. Frequency of somatic broad genomic aberrations in the “Chr7-, 8q-, 9-, 13-, 20-gain” groups, in the “Chr18-,8p-loss” groups, and in “Chr6-, 22-CNLOH” groups.

Tumor Groups according to Chr7-Gain

SB-GAINS

	Chr	1	2	3	4	5	6	7	8	9	10	11	12	13	14	15	16	17	18	19	20	21	22	X	Y
C	q/w	0	0	0	0	5	0	**0**	42	0	0	5	0	32	0	0	11	0	0	0	21	0	0	5	0
	p	0	0	0	0	11	0	**0**	0	5	0	0	0	0	0	0	11	0	0	0	0	0	0	16	0
S	q/w	18	23	45	5	9	5	**100**	68	27	5	23	9	73	9	0	27	32	0	14	86	5	9	55	14
	p	0	9	5	9	18	0	**0**	5	14	0	9	9	0	0	0	0	5	5	0	0	0	0	0	0

SB-LOSSES

	Chr	1	2	3	4	5	6	7	8	9	10	11	12	13	14	15	16	17	18	19	20	21	22	X	Y
C	q/w	0	0	0	5	0	0	0	0	0	0	0	0	0	0	5	5	0	0	26	5	0	0	5	11
	p	5	0	11	0	0	0	0	16	0	0	0	0	5	0	0	0	0	16	0	0	11	0	0	0
S	q/w	5	5	0	32	50	9	0	0	14	9	0	0	5	0	18	41	5	5	77	14	0	14	18	5
	p	36	5	5	9	0	0	0	64	5	5	0	0	23	0	0	0	5	36	0	5	18	0	0	0

C: Control CRC group with Chr7 disomy **S:** Selected CRC group with Chr7 gain **SB**: somatic broad. Numbers in the Table refer to percentages of tumor samples bearing a specific chromosomal aberration in each group.
Chr7 disomic CRC (n=19), Chr7 gain CRC (n=22), Normal Mucosae (n=26)

Tumor Groups according to Chr8q-Gain

SB-GAINS

	Chr	1	2	3	4	5	6	7	8	9	10	11	12	13	14	15	16	17	18	19	20	21	22	X	Y
**C**	**q/w**	12	6	6	0	12	0	35	**0**	0	0	12	0	41	0	0	6	0	0	0	35	0	6	24	6
	**p**	0	0	0	0	0	0	0	**0**	6	0	0	0	0	0	0	0	0	0	0	0	0	0	6	0
**S**	**q/w**	7	17	28	3	3	0	55	**100**	31	7	17	3	72	7	0	21	28	0	7	76	3	3	38	7
	**p**	0	3	0	10	0	0	7	**0**	10	0	7	7	0	0	0	7	3	3	0	0	0	0	7	0

SB-LOSSES

	Chr	1	2	3	4	5	6	7	8	9	10	11	12	13	14	15	16	17	18	19	20	21	22	X	Y
**C**	**q/w**	0	6	0	18	18	0	0	0	12	0	0	6	0	18	6	0	0	41	6	0	0	6	0	12
	**p**	18	6	6	6	0	0	0	29	0	0	0	18	0	0	0	6	12	0	0	12	0	0	0	0
**S**	**q/w**	3	0	0	17	28	10	0	0	3	10	0	0	0	10	38	3	3	66	14	0	7	14	10	3
	**p**	21	0	7	7	0	0	0	59	3	3	0	10	0	0	0	0	48	0	3	17	0	0	0	0

C: Control CRC group with Chr8q disomy **S:** Selected CRC group with Chr8q gain **SB**: somatic broad. Numbers in the Table refer to percentages of tumor samples bearing a specific chromosomal aberration in each group.
Chr8q disomic CRC (n=17), Chr8q gain CRC (n=28), Normal Mucosae (n=26)

Tumor Groups according to Chr9-Gain

SB-GAINS

	Chr	1	2	3	4	5	6	7	8	9	10	11	12	13	14	15	16	17	18	19	20	21	22	X	Y
**C**	**q/w**	7	10	7	0	3	0	34	52	**0**	0	10	0	45	0	0	14	17	0	3	48	0	7	24	3
	**p**	0	0	0	7	0	0	7	0	**0**	0	0	0	0	0	0	7	0	0	0	0	0	0	10	0
**S**	**q/w**	11	0	44	11	0	11	67	89	**100**	22	33	11	78	22	0	11	33	0	11	89	0	0	67	11
	**p**	0	22	0	0	0	0	0	11	**0**	0	11	22	0	0	0	0	0	0	0	0	0	0	0	0

SB-LOSSES

	Chr	1	2	3	4	5	6	7	8	9	10	11	12	13	14	15	16	17	18	19	20	21	22	X	Y
**C**	**q/w**	0	0	0	3	14	7	0	0	0	7	0	3	0	7	14	0	0	38	10	0	0	0	7	3
	**p**	24	3	7	7	0	0	0	31	0	0	0	3	0	0	0	3	24	0	0	10	0	0	0	0
**S**	**q/w**	11	0	0	33	33	11	0	0	0	11	0	0	0	0	33	11	11	89	11	0	33	11	0	11
	**p**	22	0	0	11	0	0	0	78	11	11	0	0	0	0	0	0	56	0	11	22	0	0	0	0

C: Control CRC group with Chr9 disomy **S:** Selected CRC group with Chr9 gain **SB**: somatic broad. Numbers in the Table refer to percentages of tumor samples bearing a specific chromosomal aberration in each group.
Chr9 disomic CRC (n=29), Chr9 gain CRC (n=9), Normal Mucosae (n=26)

Tumor Groups according to Chr13-Gain

SB-GAINS

	Chr	1	2	3	4	5	6	7	8	9	10	11	12	13	14	15	16	17	18	19	20	21	22	X	Y
**C**	**q/w**	0	13	13	0	6	0	25	44	6	0	13	0	**0**	0	0	19	13	0	0	38	0	6	13	0
	**p**	0	0	0	0	6	0	0	0	6	0	0	0	**0**	0	0	0	0	0	0	0	0	0	13	0
**S**	**q/w**	15	15	26	4	7	0	59	74	26	7	19	4	**100**	7	0	11	22	0	7	70	4	4	48	7
	**p**	0	4	0	11	15	0	7	0	11	0	7	7	**0**	0	0	7	4	4	0	0	0	0	0	0

SB-LOSSES

	Chr	1	2	3	4	5	6	7	8	9	10	11	12	13	14	15	16	17	18	19	20	21	22	X	Y
**C**	**q/w**	6	0	0	6	13	6	0	0	0	0	0	0	0	0	6	0	6	25	0	0	0	0	6	0
	**p**	6	0	6	0	0	0	0	25	0	0	0	6	0	0	0	0	19	0	6	13	0	0	0	0
**S**	**q/w**	0	4	0	22	33	7	0	0	11	11	0	4	0	22	41	4	0	74	15	0	7	19	4	11
	**p**	26	0	4	7	0	0	0	63	4	4	0	19	0	0	0	4	44	0	0	15	0	0	0	0

C: Control CRC group with Chr13 disomy **S:** Selected CRC group with Chr13 gain **SB**: somatic broad. Numbers in the Table refer to percentages of tumor samples bearing a specific chromosomal aberration in each group.
Chr13 disomic CRC (n=16), Chr13 gain CRC (n=27),Normal Mucosae (n=26)

Tumor Groups according to Chr20-Gain

SB-GAINS

	Chr	1	2	3	4	5	6	7	8	9	10	11	12	13	14	15	16	17	18	19	**20**	21	22	X	Y
**C**	**q/w**	5	0	5	0	5	0	16	37	5	5	11	0	42	0	0	0	0	0	0	**0**	0	0	11	5
	**p**	0	0	5	0	16	0	0	0	0	0	0	0	0	0	0	11	0	0	0	**0**	0	0	5	0
**S**	**q/w**	11	22	33	4	7	4	70	78	30	4	22	7	70	7	0	30	30	0	11	**100**	4	7	52	7
	**p**	0	7	0	11	11	0	7	4	15	0	7	7	0	0	0	0	4	4	0	**0**	0	0	7	0

SB-LOSSES

	Chr	1	2	3	4	5	6	7	8	9	10	11	12	13	14	15	16	17	18	19	20	21	22	X	Y
**C**	**q/w**	0	5	0	0	0	0	0	0	5	0	0	0	0	5	0	0	0	26	11	0	0	0	0	0
	**p**	11	5	5	5	0	0	0	11	0	0	0	0	0	0	0	0	16	0	0	5	0	0	0	0
**S**	**q/w**	4	0	0	30	41	11	0	0	7	11	0	4	0	19	44	4	4	81	11	0	11	19	11	15
	**p**	30	0	7	7	0	0	0	74	4	4	0	22	0	0	0	4	48	0	4	22	0	0	0	0

C: Control CRC group with Chr20 disomy **S:** Selected CRC group with Chr20 gain **SB**: somatic broad. Numbers in the Table refer to percentages of tumor samples bearing a specific chromosomal aberration in each group.
Chr20 disomic CRC (n=19), Chr20 gain CRC (n=27),Normal Mucosae (n=26)

Tumor Groups according to Chr8p-Loss

SB-GAINS

	Chr	1	2	3	4	5	6	7	8	9	10	11	12	13	14	15	16	17	18	19	20	21	22	X	Y
**C**	**q/w**	4	9	4	0	4	0	30	48	4	0	4	0	43	0	0	9	9	0	4	30	0	4	13	9
	**p**	0	0	0	0	0	0	0	0	0	0	0	0	0	0	0	9	0	4	0	0	0	0	4	0
**S**	**q/w**	16	16	32	0	11	0	58	74	32	11	26	0	79	5	0	21	26	0	5	89	5	5	53	5
	**p**	0	0	0	16	0	0	11	0	16	0	11	11	0	0	0	0	5	0	0	0	0	0	11	0

SB-LOSSES

	Chr	1	2	3	4	5	6	7	8	9	10	11	12	13	14	15	16	17	18	19	20	21	22	X	Y
**C**	**q/w**	0	0	0	9	4	0	0	**0**	4	0	0	0	0	4	9	0	0	22	13	0	0	4	0	0
	**p**	22	4	0	4	0	0	0	**0**	0	0	0	0	0	0	0	0	13	0	0	4	0	0	0	0
**S**	**q/w**	5	5	0	32	37	16	0	**0**	11	16	0	5	0	26	42	5	5	100	11	0	5	16	11	16
	**p**	16	0	16	5	0	0	0	**100**	5	5	0	26	0	0	0	5	58	0	5	32	0	0	0	0

C: Control CRC group with Chr8p disomy **S:** Selected CRC group with Chr8p loss **SB**: somatic broad. Numbers in the Table refer to percentages of tumor samples bearing a specific chromosomal aberration in each group.

Chr8p disomic CRC (n=23), Chr8p loss CRC (n=19),Normal Mucosae (n=26)

Tumor Groups according to Chr18-Loss

SB-GAINS

	Chr	1	2	3	4	5	6	7	8	9	10	11	12	13	14	15	16	17	18	19	20	21	22	X	Y
**C**	**%q/w**	0	6	0	0	6	0	28	50	6	0	0	0	39	0	0	6	11	0	0	33	0	6	11	0
	**%p**	0	0	0	0	17	0	0	0	0	0	0	0	0	0	0	11	0	0	0	0	0	0	0	0
**S**	**%q/w**	15	19	35	0	8	4	62	69	27	8	27	8	73	4	0	27	23	0	12	81	4	4	50	12
	**%p**	0	4	0	12	12	0	8	4	15	0	8	8	0	0	0	0	4	4	0	0	0	0	8	0

SB-LOSSES

	Chr	1	2	3	4	5	6	7	8	9	10	11	12	13	14	15	16	17	18	19	20	21	22	X	Y
**C**	**%q/w**	0	0	0	0	6	0	0	0	0	0	0	0	0	0	6	0	0	**0**	6	0	0	0	0	0
	**%p**	6	0	0	0	0	0	0	6	0	0	0	0	0	0	0	0	0	**0**	0	0	0	0	0	0
**S**	**%q/w**	4	4	0	35	35	12	0	0	12	12	0	4	0	23	38	4	4	**100**	15	0	8	19	12	15
	**%p**	31	4	12	8	0	0	0	77	4	4	0	23	0	0	0	4	54	**0**	4	27	0	0	0	0

C: Control CRC group with Chr18 disomy **S:** Selected CRC group with Chr18 loss **SB**: somatic broad. Numbers in the Table refer to percentages of tumor samples bearing a specific chromosomal aberration in each group.
Chr18 disomic CRC (n=18), Chr18 loss CRC (n=26),Normal Mucosae (n=26)

Tumor Groups according to Chr6-CNLOH

Somatic CNLOH

	Chr	1	2	3	4	5	6	7	8	9	10	11	12	13	14	15	16	17	18	19	20	21	22	X	Y
**C**	**%q/w**	0	3	0	3	16	**0**	0	0	13	10	10	0	0	0	0	0	10	6	3	6	3	10	0	0
	**%p**	6	6	6	0	0	**0**	6	0	0	3	0	6	0	0	0	0	6	0	0	0	0	0	0	0
**S**	**%q/w**	25	0	0	13	25	**100**	38	0	0	0	0	38	0	25	13	0	13	0	13	0	0	38	0	0
	**%p**	0	0	0	0	0	**0**	0	0	0	0	0	0	0	0	0	0	0	0	0	25	0	0	0	0

SB-GAINS

	Chr	1	2	3	4	5	6	7	8	9	10	11	12	13	14	15	16	17	18	19	20	21	22	X	Y
**C**	**q/w**	6	10	13	3	6	0	45	55	10	0	6	0	55	3	0	16	10	0	6	55	3	6	26	10
	**p**	0	3	0	3	13	0	0	0	6	0	6	3	0	0	0	6	3	3	0	0	0	0	10	0
**S**	**q/w**	13	38	38	0	13	0	50	88	38	13	50	13	88	0	0	25	13	0	0	75	0	0	63	0
	**p**	0	0	0	0	25	0	13	0	25	0	0	0	0	0	0	0	0	0	0	0	0	0	0	0

SB-LOSSES

	Chr	1	2	3	4	5	6	7	8	9	10	11	12	13	14	15	16	17	18	19	20	21	22	X	Y
**C**	**q/w**	0	0	0	23	26	0	0	0	6	0	0	3	0	13	16	3	0	48	13	0	3	13	6	10
	**p**	23	3	6	6	0	0	0	35	0	0	0	13	0	0	0	3	19	0	0	16	0	0	0	0
**S**	**q/w**	0	13	0	0	25	0	0	0	13	0	0	0	0	25	50	0	0	88	13	0	0	13	13	0
	**p**	25	0	13	13	0	0	0	88	0	0	0	25	0	0	0	0	88	0	0	13	0	0	0	0

C: Control CRC group with Chr6 disomy **S:** Selected CRC group with Chr6 CNLOH **SB**: somatic broad. Numbers in the Table refer to percentages of tumor samples bearing a specific chromosomal aberration in each group.
Chr6 disomic CRC (n=31), Chr6 CNLOH CRC(n=8),Normal Mucosae (n=26)

Tumor Groups according to Chr22-CNLOH

Somatic CNLOH

	Chr	1	2	3	4	5	6	7	8	9	10	11	12	13	14	15	16	17	18	19	20	21	22	X	Y
**C**	**q/w**	3	0	0	3	19	13	9	3	16	6	3	3	3	9	3	0	6	3	6	3	0	**0**	0	0
	**p**	0	6	3	0	0	0	0	0	0	6	0	0	0	0	0	0	3	0	0	3	0	**0**	0	0
**S**	**q/w**	0	17	0	17	0	50	0	0	0	0	33	17	0	0	0	0	0	0	17	0	0	**100**	0	0
	**p**	0	0	0	0	0	0	0	0	0	0	0	33	0	0	0	0	0	0	0	0	0	**0**	0	0

SB-GAINS

	Chr	1	2	3	4	5	6	7	8	9	10	11	12	13	14	15	16	17	18	19	20	21	22	X	Y
**C**	**q/w**	6	3	19	0	3	3	41	53	22	6	16	3	53	3	0	9	19	0	6	53	0	0	34	3
	**p**	0	3	0	6	16	0	3	3	3	0	0	3	0	0	0	6	0	0	0	0	0	0	6	0
**S**	**q/w**	17	17	33	17	17	0	50	83	17	0	17	0	67	17	0	0	17	0	0	50	0	0	67	0
	**p**	0	17	0	0	0	0	17	0	0	0	0	0	0	0	0	0	0	0	0	0	0	0	0	0

SB-LOSSES

	Chr	1	2	3	4	5	6	7	8	9	10	11	12	13	14	15	16	17	18	19	20	21	22	X	Y
**C**	**q/w**	3	0	0	16	19	9	0	0	3	9	0	0	0	0	13	0	3	53	6	0	6	0	3	0
	**p**	16	3	3	3	0	0	0	41	3	3	0	9	0	0	0	0	31	0	3	16	0	0	0	0
**S**	**q/w**	0	17	0	0	17	0	0	0	17	0	0	0	0	33	50	0	0	50	17	0	17	0	0	0
	**p**	50	0	17	33	0	0	0	50	0	0	0	0	0	0	0	0	50	0	0	0	0	0	0	0

C: Control CRC group with Chr22 disomy **S:** Selected CRC group with Chr22 CNLOH **SB**: somatic broad. Numbers in the Table refer to percentages of tumor samples bearing a specific chromosomal aberration in each group.
Chr22 disomic CRC (n=32), Chr22 CNLOH CRC(n=6),Normal Mucosae (n=26)

Supplementary Table S3. Transcript classes defined on the basis of four indices (FC1, FC2, FC3, FC4), three CRC groups (All CRC, Control CRC, Selected CRC) and normal colonic mucosae group (Normal)**.**

**Transcript Class**	**Abbreviations**	**FC1** All CRC vs Normal	**FC2** Selected CRC vs Control CRC	**FC3** Control CRC vs Normal	**FC4** Selected CRC vs Normal
Upregulated	UpT	**>1.5**			
Downregulated	DownT	**<-1.5**			
Overexpressed	OverT		**>1.3**		
Underexpressed	UnderT		**<-1.3**		
Upregulated	UpT			**>1.5**	
Downregulated	DownT			**<-1.5**	
Upregulated	UpT				**>1.5**
Downregulated	DownT				**<-1.5**
Overexpressed and Upregulated	Over-UpT		**>1.3**	**>1.5**	
Underexpressed and Upregulated	Under-UpT		**<-1.3**	**>1.5**	
Overexpressed and Downregulated	Over-DownT		**>1.3**	**<-1.5**	
Underexpressed and Downregulated	Under-DownT		**<-1.3**	**<-1.5**	
Transcripts with positive fold-change	PositiveT			**>1**	
Transcripts with negative fold-change	NegativeT			**<-1**	
Overexpresssed PositiveT	Over-PositiveT		**>1.3**	**>1**	
Overexpresssed NegativeT	Over-NegativeT		**>1.3**	**<-1**	

Supplementary Table S4. List of Over-UpT genes (only protein coding genes) in Chr20, Chr8q, Chr13, Chr7, Chr9.

Over UpT in Chr 20

Transcript Cluster ID	FDR p-value	FC3	FC4	FC2	Gene Symbol	Description	Known cancer related gene	RNA-Seq confirmed Over-UpT
TC20000384.hg.1	1.24E-09	15.41	43.2	2.8	CSE1L	CSE1 chromosome segregation 1-like (yeast);		Yes
TC20000208.hg.1	1.02E-08	1.74	4.77	2.74	POFUT1	protein O-fucosyltransferase 1; microRNA 1825;		Yes
TC20000940.hg.1	3.91E-10	3.35	8.95	2.67	DPM1	dolichyl-phosphate mannosyltransferase polypeptide 1, catalytic subunit;		Yes
TC20000198.hg.1	2.77E-10	5.14	13.63	2.65	TPX2	TPX2, microtubule-associated, homolog (Xenopus laevis);	SSC-820	Yes
TC20000271.hg.1	6.78E-09	13.98	36.85	2.64	RPN2	ribophorin II;		Yes
TC20000076.hg.1	0.000001	5.08	12.1	2.39	PLCB4	phospholipase C, beta 4;	CG-299	Yes

TC20001761.hg.1	7.15E-10	3.15	7.47	2.37	CPNE1	copine I;		Yes
TC20000822.hg.1	1.55E-09	2.08	4.76	2.29	RBL1	retinoblastoma-like 1 (p107);		Yes
TC20000382.hg.1	0.000002	2.11	4.67	2.21	ARFGEF2	ADP-ribosylation factor guanine nucleotide-exchange factor 2 (brefeldin A-inhibited);		No
TC20000779.hg.1	6.13E-10	3.98	8.77	2.2	EIF2S2	eukaryotic translation initiation factor 2, subunit 2 beta, 38kDa;	SSC-820	Yes
TC20000780.hg.1	8.36E-10	2.7	5.74	2.13	AHCY	adenosylhomocysteinase;		Yes
TC20000216.hg.1	1.18E-07	3.26	6.77	2.08	MAPRE1	microtubule-associated protein, RP/EB family, member 1;		Yes
TC20000207.hg.1	1.89E-08	3.21	6.6	2.06	TM9SF4	transmembrane 9 superfamily protein member 4;		Yes
TC20000467.hg.1	2.05E-10	1.85	3.73	2.01	NELFCD	negative elongation factor complex		Yes

						member C/D;		
TC20000878.hg.1	0.000027	3.34	6.73	2.01	SDC4	syndecan 4;	CG-719	Yes
TC20001754.hg.1	0.000036	2.22	4.46	2.01	SLMO2	slowmo homolog 2 (Drosophila);		No
TC20000784.hg.1	1.70E-08	2.28	4.52	1.98	PIGU	phosphatidylinositol glycan anchor biosynthesis, class U;		Yes
TC20000489.hg.1	4.05E-09	3.46	6.75	1.95	RPS21	ribosomal protein S21;		Yes
TC20001753.hg.1	3.15E-08	1.74	3.38	1.94	ATP5E	ATP synthase, H+ transporting, mitochondrial F1 complex, epsilon subunit;		No
TC20001738.hg.1	1.05E-09	2.04	3.93	1.93	PCMTD2	protein-L-isoaspartate (D-aspartate) O-methyltransferase domain containing 2;		Yes
TC20000791.hg.1	0.000004	1.53	2.92	1.91	TRPC4AP	transient receptor potential cation channel, subfamily C, member 4 associated protein;		Yes

TC20000393.hg.1	3.22E-08	4.62	8.71	1.89	RNF114	ring finger protein 114;		Yes
TC20001730.hg.1	0.000001	1.59	2.98	1.88	C20orf24	chromosome 20 open reading frame 24;		Yes
TC20000789.hg.1	2.16E-07	1.73	3.11	1.8	GSS	glutathione synthetase;		Yes
TC20000786.hg.1	5.12E-07	1.77	3.16	1.79	NCOA6	nuclear receptor coactivator 6;		No
TC20000337.hg.1	2.99E-07	2.68	4.78	1.78	YWHAB	tyrosine 3-monooxygenase/tryptophan 5-monooxygenase activation protein, beta polypeptide;		No
TC20001005.hg.1	9.44E-09	2.48	4.39	1.77	PSMA7	proteasome (prosome, macropain) subunit, alpha type, 7;		Yes
TC20000603.hg.1	6.60E-08	9.07	15.52	1.71	FERMT1	fermitin family member 1;		No
TC20001747.hg.1	3.36E-07	1.95	3.26	1.67	EIF6	eukaryotic translation initiation factor 6;		Yes

TC20000928.hg.1	0.001357	2.23	3.72	1.67	B4GALT5	UDP-Gal:betaGlcNAc beta 1,4-galactosyltransferase, polypeptide 5;		No
TC20000818.hg.1	4.19E-09	1.75	2.87	1.64	DSN1	DSN1, MIND kinetochore complex component, homolog (S. cerevisiae);		Yes
TC20000805.hg.1	4.26E-08	1.75	2.85	1.63	RBM39	RNA binding motif protein 39;		No
TC20000385.hg.1	2.84E-10	1.53	2.47	1.62	DDX27	DEAD (Asp-Glu-Ala-Asp) box polypeptide 27;		Yes
TC20000871.hg.1	8.04E-09	5.25	8.34	1.59	TOMM34	translocase of outer mitochondrial membrane 34;		Yes
TC20001758.hg.1	1.18E-08	1.53	2.43	1.59	RTFDC1	replication termination factor 2 domain containing 1;		No
TC20000829.hg.1	2.07E-08	2.66	4.2	1.58	TTI1	TELO2 interacting protein 1;		Yes
TC20000320.hg.1	1.55E-08	2.15	3.29	1.53	MYBL2	v-myb myelobla		Yes

						stosis viral oncogene homolog (avian)-like 2;		
TC20000893.hg.1	0.000001	1.89	2.87	1.52	PLTP	phospholipid transfer protein;		No
TC20000285.hg.1	0.000001	2.7	4.06	1.51	RALGAPB	Ral GTPase activating protein, beta subunit (non-catalytic);		No
TC20001762.hg.1	6.53E-10	3.96	5.89	1.49	RBM12	RNA binding motif protein 12; copine I;		No
TC20001733.hg.1	1.02E-07	1.53	2.27	1.49	STX16	syntaxin 16;		Yes
TC20000357.hg.1	5.79E-09	1.83	2.66	1.46	UBE2C	ubiquitin-conjugating enzyme E2C;		Yes
TC20000526.hg.1	1.47E-07	1.88	2.74	1.46	TPD52L2	tumor protein D52-like 2;		Yes
TC20001026.hg.1	3.32E-09	2.85	4.12	1.45	YTHDF1	YTH domain family, member 1;		Yes
TC20000458.hg.1	2.45E-08	1.65	2.39	1.45	VAPB	VAMP (vesicle-associated membrane protein)-associate		No

						d protein B and C;		
TC20000 896.hg.1	1.13E-07	1.87	2.71	1.45	NCOA5	nuclear receptor coactivat or 5;		Yes
TC20000 040.hg.1	1.14E-08	2.11	3.02	1.43	CDC25B	cell division cycle 25B;		No
TC20000 191.hg.1	1.57E-09	2.3	3.25	1.42	HM13	histocom patibility (minor) 13; malignan t T cell amplified sequence 1 pseudog ene;		Yes
TC20000 923.hg.1	0.000001	3.53	5.01	1.42	STAU1	staufen double-stranded RNA binding protein 1; staufen, RNA binding protein, homolog 1 (Drosoph ila);		Yes
TC20000 457.hg.1	0.000002	2.24	3.17	1.42	RAB22A	RAB22A, member RAS oncogen e family;		No
TC20000 501.hg.1	5.56E-07	1.97	2.71	1.38	GID8	GID complex subunit 8 homolog (S. cerevisia e); chromos ome 20 open reading		Yes

						frame 11;		
TC20000339.hg.1	0.000001	1.53	2.09	1.37	STK4	serine/threonine kinase 4;		Yes
TC20000372.hg.1	0.000072	2.09	2.86	1.37	NCOA3	nuclear receptor coactivator 3;	SCC-820	No
TC20000347.hg.1	6.13E-07	3.14	4.26	1.36	PIGT	phosphatidylinositol glycan anchor biosynthesis, class T;		No
TC20000140.hg.1	0.000003	3.9	5.3	1.36	XRN2	5'-3' exoribonuclease 2;		Yes
TC20000316.hg.1	1.62E-08	2.85	3.81	1.34	SRSF6	serine/arginine-rich splicing factor 6;		No
TC20000939.hg.1	7.15E-10	2.18	2.88	1.32	ADNP	activity-dependent neuroprotector homeobox;	SCC-820	Yes
TC20000302.hg.1	0.000002	4.01	5.3	1.32	TOP1	topoisomerase (DNA) I;	CG-719; SCC-820	No
TC20000657.hg.1	0.000828	1.77	2.34	1.32	RRBP1	ribosome binding protein 1;		No

Over-UpT genes in Chr8q

Transcript Cluster ID	FDR p-value	FC3	FC4	FC2	Gene Symbol	Description	Known Cancer related Gene	RNA-Seq confirmed Over-UpT
TC08001191.hg.1	8.5E-09	13.73	30.53	2.22	PRKDC	protein kinase, DNA-activated, catalytic polypeptide;	SCC-820	Yes
TC08000729.hg.1	0.000003	2.87	5.26	1.83	SQLE	squalene epoxidase;		Yes
TC08001524.hg.1	7.37E-09	5.34	9.57	1.79	EIF3E	eukaryotic translation initiation factor 3, subunit E;	CG-719	Yes
TC08001545.hg.1	0.000000022	4.31	7.67	1.78	EIF3H	eukaryotic translation initiation factor 3, subunit H;		Yes
TC08001501.hg.1	0.000000116	4.73	8.28	1.75	AZIN1	antizyme inhibitor 1;		No
TC08000663.hg.1	0.000000875	2.15	3.76	1.75	EMC2	ER membrane protein complex subunit 2;		No
TC08001584.hg.1	0.000000082	3.56	6.14	1.73	ATAD2	ATPase family, AAA domain		No

						containing 2;		
TC08001616.hg.1	0.000007	3.07	5.1	1.66	KIAA0196	KIAA0196;		No
TC08001210.hg.1	0.000274	2.19	3.64	1.66	RB1CC1	RB1-inducible coiled-coil 1;		No
TC08000528.hg.1	0.000011	2.62	4.23	1.61	LRRCC1	leucine rich repeat and coiled-coil centrosomal protein 1;		No
TC08000594.hg.1	4.43E-08	10.11	16.12	1.59	MTDH	metadherin;		No
TC08001264.hg.1	4.77E-08	2.84	4.45	1.57	ASPH	aspartate beta-hydroxylase;		No
TC08001312.hg.1	0.000016	4.48	6.99	1.56	TRAM1	translocation associated membrane protein 1;		No
TC08001458.hg.1	4.85E-08	3.39	5.16	1.52	RPL30	ribosomal protein L30;		Yes
TC08000648.hg.1	0.000000367	3.07	4.65	1.52	DCAF13	DDB1 and CUL4 associated factor 13;		Yes
TC08001293.hg.1	0.000054	2.5	3.79	1.52	ARFGEF1	ADP-ribosylation factor guanine nucleotide-exchange factor 1 (brefeldin A-inhibited);		No

TC08000695.hg.1	0.000315	1.76	2.64	1.5	MAL2	mal, T-cell differentiation protein 2 (gene/pseudogene);		No
TC08001497.hg.1	0.000003	2.41	3.55	1.47	UBR5	ubiquitin protein ligase E3 component n-recognin 5;	CGC-719;SSC-819	No
TC08000814.hg.1	0.000023	1.59	2.34	1.47	LY6E	lymphocyte antigen 6 complex, locus E;		No
TC08001684.hg.1	0.00000032	1.77	2.59	1.46	AGO2	argonaute RISC catalytic component 2;		No
TC08000641.hg.1	0.000018	2.86	4.18	1.46	ATP6V1C1	ATPase, H+ transporting, lysosomal 42kDa, V1 subunit C1;		Yes
TC08000575.hg.1	0.00004	2.78	4.07	1.46	ESRP1	epithelial splicing regulatory protein 1;		No
TC08001762.hg.1	8.47E-09	17.52	25.38	1.45	RPL8	ribosomal protein L8		Yes
TC08000588.hg.1	4.42E-08	4.08	5.93	1.45	PTDSS1	phosphatidylserine synthase 1;		Yes
TC08000733.hg.1	0.009959	1.53	2.22	1.45	TRIB1	tribbles homolog 1 (Drosophila);		No

TC08001528.hg.1	5.22E-09	2.42	3.48	1.44	NUDCD1	NudC domain containing 1;		Yes
TC08000456.hg.1	0.000455	2.59	3.67	1.42	SULF1	sulfatase 1;		No
TC08001723.hg.1	9.02E-08	2.19	3.1	1.41	TSTA3	tissue specific transplantation antigen P35B		No
TC08000538.hg.1	0.000006	3.01	4.25	1.41	WWP1	WW domain containing E3 ubiquitin protein ligase 1;		No
TC08000403.hg.1	0.000053	2.43	3.44	1.41	SDCBP	syndecan binding protein (syntenin);		No
TC08000539.hg.1	0.000012	3.58	4.96	1.39	CPNE3	copine III;		No
TC08000720.hg.1	0.000001	3.55	4.9	1.38	FAM91A1	family with sequence similarity 91, member A1;		Yes
TC08001218.hg.1	0.000002	2.73	3.75	1.38	LYPLA1	lysophospholipase I;		Yes
TC08000545.hg.1	0.000002	2.87	3.98	1.38	RIPK2	receptor-interacting serine-threonine kinase 2;		No
TC08000346.hg.1	0.000000387	2.36	3.23	1.37	MCM4	minichromosome maintenance complex component 4;		No
TC08001575.hg.1	0.000015	2.42	3.31	1.37	DERL1	derlin 1;		No

TC08001546.hg.1	0.000000807	2.79	3.71	1.33	RAD21	RAD21 homolog (S. pombe); microRNA 3610;	CG-299; CGC-719; SCC-820	No
TC08001216.hg.1	0.000000813	1.73	2.3	1.33	TCEA1	transcription elongation factor A (SII), 1;	CGC-719	No
TC08001269.hg.1	0.000003	2.29	3.05	1.33	GGH	gamma-glutamyl hydrolase (conjugase, folylpoly gammaglutamyl hydrolase);		Yes
TC08000577.hg.1	0.000013	1.87	2.45	1.31	DPY19L4	dpy-19-like 4 (C. elegans);		Yes
TC08000597.hg.1	0.000016	3.41	4.48	1.31	LAPTM4B	lysosomal protein transmembrane 4 beta;		Yes

Over-UpT genes in Chr13

Transcript Cluster ID	FDR p-value	FC3	FC4	FC2	Gene Symbol	Description	Known Cancer related Gene	RNA-Seq confirmed Over-UpT
TC13000491.hg.1	0.000611	2.96	6.83	2.3	PARP4	poly (ADP-ribose) polymerase family, member 4;	SCC-820	No
TC13000285.hg.1	1.05E-07	3.88	8.53	2.2	UCHL3	ubiquitin carboxyl-terminal esterase L3 (ubiquitin thiolesterase);		Yes
TC13000539.hg.1	1.78E-07	7.32	14.13	1.93	HSPH1	heat shock 105kDa/110kDa protein 1;		Yes
TC13000356.hg.1	0.000008	9.05	17.47	1.93	TM9SF2	transmembrane 9 superfamily member 2;		No
TC13000336.hg.1	0.000004	5.62	10.68	1.9	DNAJC3	DnaJ (Hsp40) homolog, subfamily C, member 3;		No
TC13000077.hg.1	6.45E-08	5.92	11.04	1.87	GTF3A	general transcription factor IIIA;		Yes

TC13000663.hg.1	0.000003	4.46	8.23	1.84	KPNA3	karyopherin alpha 3 (importin alpha 4);		No
TC13000344.hg.1	0.000023	1.93	3.48	1.8	FARP1	FERM, RhoGEF (ARHGEF) and pleckstrin domain protein 1 (chondrocyte-derived); FARP1 intronic transcript 1 (non-protein coding);		Yes
TC13000506.hg.1	0.00051	2.28	4.11	1.8	USP12	ubiquitin specific peptidase 12;		No
TC13000472.hg.1	0.000007	5.03	8.53	1.7	ZDHHC20	zinc finger, DHHC-type containing 20;		No
TC13000647.hg.1	0.000002	3.34	5.65	1.69	ESD	esterase D;		No
TC13000226.hg.1	7.34E-07	2.83	4.7	1.66	SUGT1	SGT1, suppressor of G2 allele of SKP1 (S. cerevisiae);		No
TC13000727.hg.1	5.89E-08	3.03	4.94	1.63	DIS3	DIS3 mitotic control homolog (S. cerevisiae);	SCC-820	No
TC13000068.hg.1	0.000008	3.11	5.07	1.63	CDK8	cyclin-dependent kinase 8;	SCC-820	Yes

TC13000112.hg.1	0.000018	2.44	3.93	1.61	PDS5B	PDS5, regulator of cohesion maintenance, homolog B (S. cerevisiae);	CG-299	No
TC13000223.hg.1	1.14E-08	2.26	3.6	1.59	CKAP2	cytoskeleton associated protein 2;		No
TC13000793.hg.1	1.77E-07	1.81	2.84	1.57	TGDS	TDP-glucose 4,6-dehydratase;		No
TC13000091.hg.1	1.50E-08	2.92	4.55	1.56	POMP	proteasome maturation protein;		Yes
TC13000330.hg.1	7.97E-07	2.05	3.17	1.55	GPR180	G protein-coupled receptor 180;		No
TC13000367.hg.1	3.58E-08	3.18	4.9	1.54	TPP2	tripeptidyl peptidase II;		No
TC13001720.hg.1	7.11E-07	1.63	2.49	1.53	BIVM	basic, immunoglobulin-like variable motif containing;		Yes
TC13000662.hg.1	2.11E-08	3.41	5.17	1.52	EBPL	emopamil binding protein-like;		No
TC13000892.hg.1	2.53E-10	1.96	2.95	1.51	PCID2	PCI domain containing 2;		Yes
TC13000411.hg.1	1.74E-09	2.32	3.49	1.51	ATP11A	ATPase, class VI,		Yes

						type 11A;		
TC13000523.hg.1	1.63E-08	4.26	6.42	1.51	SLC7A1	solute carrier family 7 (cationic amino acid transporter, y+ system), member 1;		Yes
TC13000421.hg.1	9.10E-07	2.8	4.23	1.51	LAMP1	lysosomal-associated membrane protein 1;	SCC-820	No
TC13000653.hg.1	0.000002	1.87	2.83	1.51	MED4	mediator complex subunit 4;		No
TC13000343.hg.1	1.78E-08	2.61	3.91	1.5	IPO5	importin 5;		Yes
TC13000111.hg.1	1.74E-07	2.01	3.03	1.5	BRCA2	breast cancer 2, early onset;	CG-299; CGC-719; SCC-820	No
TC13000461.hg.1	3.34E-09	2.79	4.13	1.48	XPO4	exportin 4;		No
TC13000807.hg.1	2.87E-08	2	2.96	1.48	UGGT2	UDP-glucose glycoprotein glucosyltransferase 2;		Yes
TC13000584.hg.1	8.71E-08	6.93	10.29	1.48	PROSER1	proline and serine rich 1;		Yes
TC13000887.hg.1	1.14E-07	1.74	2.56	1.48	TUBGCP3	tubulin, gamma complex associated protein 3;		Yes
TC13000484.hg.1	4.02E-07	1.86	2.75	1.48	MIPEP	mitochondrial		Yes

						intermediate peptidase;		
TC13000701.hg.1	4.33E-08	1.86	2.72	1.46	DIAPH3	diaphanous homolog 3 (Drosophila);		No
TC13000426.hg.1	7.88E-08	2.33	3.42	1.46	TFDP1	transcription factor Dp-1;		Yes
TC13000340.hg.1	0.000029	3.45	5	1.45	MBNL2	muscleblind-like splicing regulator 2;		No
TC13000064.hg.1	3.66E-08	2.92	4.22	1.44	NUPL1	nucleoporin like 1;		No
TC13000271.hg.1	3.10E-07	1.7	2.44	1.44	PIBF1	progesterone immunomodulatory binding factor 1;		No
TC13000152.hg.1	0.000001	3.19	4.58	1.44	AKAP11	A kinase (PRKA) anchor protein 11;		No
TC13000633.hg.1	5.51E-07	12.08	17.32	1.43	RCN1P2	reticulocalbin 1, EF-hand calcium binding domain pseudogene 2		No
TC13000083.hg.1	0.000004	1.82	2.6	1.43	ATP5EP2	ATP synthase, H+ transporting, mitochondrial F1 complex, epsilon subunit		Yes

						pseudogene 2;		
TC13000644.hg.1	0.000468	1.58	2.26	1.43	KIAA0226L	KIAA0226-like;		Yes
TC13000534.hg.1	5.84E-09	3.23	4.59	1.42	HMGB1	high mobility group box 1;		No
TC13000435.hg.1	8.28E-09	2.42	3.42	1.41	CDC16	cell division cycle 16;		Yes
TC13000130.hg.1	9.36E-07	2.31	3.26	1.41	UFM1	ubiquitin-fold modifier 1;		No
TC13000626.hg.1	3.34E-09	2.35	3.28	1.4	NUFIP1	nuclear fragile X mental retardation protein interacting protein 1;		Yes
TC13000468.hg.1	9.21E-07	1.98	2.75	1.39	SKA3	spindle and kinetochore associated complex subunit 3;		No
TC13000879.hg.1	0.000005	3.49	4.82	1.38	ANKRD10-IT1	ANKRD10 intronic transcript 1 (non-protein coding);		No
TC13000176.hg.1	0.000018	1.99	2.75	1.38	COG3	component of oligomeric golgi complex 3;		No
TC13000419.hg.1	2.69E-08	1.75	2.4	1.37	CUL4A	cullin 4A;	SCC-820	Yes
TC13000513.hg.1	0.000229	1.93	2.65	1.37	CDX2	caudal type homeobox 2;	CGC-719	No

TC13000273.hg.1	0.000318	3.27	4.47	1.37	KLF5	Kruppel-like factor 5 (intestinal);	CG-299	No
TC13000099.hg.1	8.17E-08	1.77	2.38	1.34	USPL1	ubiquitin specific peptidase like 1;		Yes
TC13000171.hg.1	1.65E-07	2.87	3.84	1.34	GTF2F2	general transcription factor IIF, polypeptide 2, 30kDa;		Yes
TC13000715.hg.1	3.93E-07	3.47	4.66	1.34	HNRNPA3P5	heterogeneous nuclear ribonucleoprotein A3 pseudogene 5		No
TC13000652.hg.1	0.000013	1.66	2.22	1.34	SUCLA2	succinate-CoA ligase, ADP-forming, beta subunit;		No
TC13000309.hg.1	2.10E-08	15.49	20.61	1.33	PTMAP5	Prothymosin, Alpha Pseudogene 5		No
TC13000524.hg.1	0.007732	2.97	3.92	1.32	UBL3	ubiquitin-like 3;		No
TC13000570.hg.1	4.26E-08	1.96	2.56	1.31	ALG5	ALG5, dolichyl-phosphate beta-glucosyltransferase;		Yes
TC13000511.hg.1	0.000246	1.83	2.4	1.31	LNX2	ligand of numb-protein X 2;		No

OverUpT in Chr7

Transcript Cluster ID	FDR p-value	FC3	FC4	FC2	Gene Symbol	Description	Known Cancer related Gene	RNA-Seq confirmed Over-UpT
TC07001182.hg.1	0.000000357	2.68	5.28	1.97	MACC1	metastasis associated in colon cancer 1;		No
TC07000726.hg.1	0.000305	4.12	5.84	1.42	CFTR	cystic fibrosis transmembrane conductance regulator (ATP-binding cassette sub-family C. member 7);		No
TC07000728.hg.1	1.13E-08	1.67	2.37	1.42	NAA38	N(alpha)-acetyltransferase 38, NatC auxiliary subunit;		Yes
TC07001373.hg.1	1.00E-05	1.52	2.16	1.42	DDC	dopa decarboxylase (aromatic L-amino acid decarboxylase);		No

TC07000248.hg.1	0.00000028	1.91	2.69	1.41	YAE1D1	Yae1 domain containing 1;		Yes
TC07001499.hg.1	7.71E-08	3.93	5.45	1.39	BAZ1B	bromodomain adjacent to zinc finger domain, 1B;		Yes
TC07001734.hg.1	1.51E-08	2.93	3.85	1.31	PUS7	pseudouridylate synthase 7 homolog (S. cerevisiae);		Yes

OverUpT in Chr9

Transcript Cluster ID	FDR p-value	FC3	FC4	FC2	Gene Symbol	Description	Known Cancer related Gene
TC09001490.hg.1	0.000001	1.66	2.7	1.63	SUSD1	sushi domain containing 1;	
TC09000099.hg.1	7.66E-07	2.47	3.87	1.56	FOCAD	focadhesin;	
TC09000874.hg.1	1.33E-09	3.97	5.79	1.46	KIAA0020	KIAA0020;	
TC09002890.hg.1	5.89E-09	4.37	6.4	1.46	TOMM5	translocase of outer mitochondrial membrane 5 homolog (yeast);	
TC09000415.hg.1	4.88E-08	6.11	8.91	1.46	CKS2	CDC28 protein kinase regulatory subunit 2;	
TC09000864.hg.1	1,65E-08	2.41	3.5	1.45	CBWD1	COBW domain containing 1; COBW domain-containing protein 1-like; COBW domain containing 5;	

TC09000697.hg.1	8.64E-10	4.66	6.7	1.44	SET	SET nuclear oncogene;	CGC-719
TC09000308.hg.1	3.85E-08	2.58	3.72	1.44	CBWD3	COBW domain containing 3; COBW domain-containing protein 5-like; COBW domain-containing protein 1-like; COBW domain containing 7; COBW domain containing 5;	CG-299
TC09000247.hg.1	7.29E-08	2.69	3.84	1.43	LOC101060578	COBW domain-containing protein 5-like;	
TC09002880.hg.1	3.13E-07	2.89	4.14	1.43	MTAP	methylthioadenosine phosphorylase;	
TC09000358.hg.1	6.43E-07	5.66	8.12	1.43	PSAT1	phosphoserine aminotransferase 1;	
TC09000417.hg.1	0.000009	1.94	2.75	1.42	SECISBP2	SECIS binding protein 2;	
TC09000781.hg.1	6.40E-09	3.18	4.47	1.4	WDR5	WD repeat domain 5;	
TC09001007.hg.1	8.03E-07	4.21	5.85	1.39	SMU1	smu-1 suppressor of mec-8	

						and unc-52 homolog (C. elegans);	
TC09000740.hg.1	6,03E-10	2.07	2.83	1.37	EXOSC2	exosome component 2;	
TC09001181.hg.1	4.22E-08	2.45	3.32	1.36	CBWD5	COBW domain containing 5; COBW domain containing 3; COBW domain-containing protein 5-like; COBW domain containing 7;	
TC09000893.hg.1	0.000024	2.44	3.31	1.36	ERMP1	endoplasmic reticulum metallopeptidase 1;	
TC09000925.hg.1	0.000501	1.77	2.4	1.36	PSIP1	PC4 and SFRS1 interacting protein 1;	CG-299; CGC-719; SCC-820
TC09001170.hg.1	2.71E-08	2.24	3.03	1.35	CBWD6	COBW domain containing 6;	
TC09000218.hg.1	0.000003	2.22	2.95	1.33	DCAF10	DDB1 and CUL4 associated factor 10;	
TC09000502.hg.1	5.65E-08	2.31	3.02	1.31	SEC61B	Sec61 beta subunit;	

Supplementary Table S5 The number of Over-UpT present in each selected chromosomes of the five gain groups (Chr20, Chr8q, Chr13, Chr7 and Chr9), the number of cancer driver genes of each list (CGC-719, SCC-820, CG-299) present on the same chromosomes and the number of cancer genes of each list belonging to the Over-UpT class; the names of such genes are reported in Supplementary Table S4.

	Chr20	Chr8q	Chr13	Chr7	Chr9	Total
**N coding transcripts per chromosome**	1068	1656	901	2138	1802	7565
**Over-UpT**	59	41	61	7	21	189
**CGC-719 (Cancer Gene Census)**	14	22	13	36	31	116
**CGC-719 Over_UpT**	2	4	2	0	2	10
**fold-enrichment**	2.59	7.34	2.27	0	6.86	3.45
**p-value**	0.1785	0.0017	0.2180	-	0.0330	0.0006
**SCC-820 (Ohshima et al 2017)**	22	14	19	35	30	120
**SCC-820 Over-UpT**	5	3	6	0	1	15
**fold-enrichment**	4.11	8.66	4.66	0	2.86	5.00
**p-value**	0.0055	0.0042	0.001	-	0.2985	2.51E-07
**CG-299 (Bailey et al 2018)**	6	4	9	15	17	51
**CG-299 Over-UpT**	1	1	3	0	2	7
**fold-enrichment**	3.02	10.10	4.92	0	10.10	5.49
**p-value**	0.2895	0.0955	0.0185	-	0.0158	0.0002

In red significant values p<0.05

Supplementary Table S6. List of Under-UpT genes (only protein coding genes) in Chr8p, Chr18.

Under-UpT in Chr8p

Transcript Cluster ID	FDR p-value (All Conditions)	Fold Change (Chr8p-disomic CRC vs. normal colon)	Fold Change (linear) (Chr8p-loss CRC vs. normal colon)	Fold Change (linear) (Chr8p-loss CRC vs. Chr8p-disomic CRC)	Gene Symbol	Description
TC08002590.hg.1	0.000283	1.53	-1.6	-2.45	DOCK5	dedicator of cytokinesis 5; NULL
TC08001015.hg.1	0.000021	2.95	1.58	-1.86	ASAH1	N-acylsphingosine amidohydrolase (acid ceramidase) 1; NULL
TC08001105.hg.1	0.000627	3.66	2.04	-1.79	TMEM66	transmembrane protein 66; NULL
TC08002612.hg.1	0.000018	1.83	1.03	-1.78	ENTPD4	ectonucleoside triphosphate diphosphohydrolase 4; NULL
TC08000135.hg.1	0.000005	3.04	1.72	-1.77	PCM1	pericentriolar material 1; NULL
TC08000298.hg.1	3.21E-07	7.07	4.19	-1.69	ADAM9	ADAM metallopeptidase domain 9; NULL
TC08001049.hg.1	1.38E-10	1.95	1.22	-1.6	TNFRSF10B	tumor necrosis factor receptor superfamily, member 10b; NULL
TC08001011.hg.1	6.96E-07	2.95	1.88	-1.57	CNOT7	CCR4-NOT transcription complex, subunit 7; NULL
TC08000213.hg.1	0.000077	2.11	1.35	-1.55	ESCO2	establishment of sister chromatid cohesion N-acetyltransferase 2; NULL
TC08001084.hg.1	0.000005	2.5	1.63	-1.53	CCDC25	coiled-coil domain containing 25; NULL
TC08000151.hg.1	0.000005	2.28	1.49	-1.53	ATP6V1B2	ATPase, H+ transporting, lysosomal 56/58kDa, V1 subunit B2; NULL
TC08001110.hg.1	0.000005	3.47	2.32	-1.49	GSR	glutathione reductase; NULL
TC08000146.hg.1	0.000015	1.54	1.03	-1.49	SH2D4A	SH2 domain containing 4A; NULL
TC08002591.hg.1	0.000002	1.73	1.17	-1.48	PPP2R2A	protein phosphatase 2, regulatory subunit B, alpha; NULL
TC08000980.hg.1	0.000002	3.62	2.49	-1.45	CTSB	cathepsin B; NULL
TC08000200.hg.1	0.000018	2.06	1.42	-1.45	BNIP3L	BCL2/adenovirus E1B 19kDa interacting protein 3-like; NULL
TC08000148.hg.1	5.86E-07	1.92	1.35	-1.42	INTS10	integrator complex subunit 10; NULL

TC08000943.hg.1	0.000013	1.76	1.24	-1.41	MFHAS1	malignant fibrous histiocytoma amplified sequence 1; NULL
TC08000258.hg.1	3.92E-07	2.13	1.55	-1.37	MAK16	MAK16 homolog (S. cerevisiae); NULL
TC08000160.hg.1	4.91E-07	3.61	2.64	-1.37	XPO7	exportin 7; NULL
TC08002578.hg.1	0.000016	1.86	1.36	-1.37	LEPROTL1	leptin receptor overlapping transcript-like 1; NULL
TC08000126.hg.1	0.000043	1.69	1.24	-1.37	VPS37A	vacuolar protein sorting 37 homolog A (S. cerevisiae); NULL
TC08000175.hg.1	3.96E-07	1.7	1.26	-1.35	KIAA1967	KIAA1967; NULL
TC08000196.hg.1	0.000008	1.95	1.44	-1.35	CDCA2	cell division cycle associated 2; NULL
TC08002579.hg.1	0.000113	1.61	1.22	-1.32	DCTN6	dynactin 6; NULL

Under-UpT in Chr18

Transcript Cluster ID	FDR p-value (All Conditions)	Fold Change (Chr18-disomic CRC vs. normal colon)	Fold Change (Chr18-loss CRC vs. normal colon)	Fold Change (Chr18-loss CRC vs. Chr18-disomic CRC)	Gene Symbol	Description
TC18000226.hg.1	1.32E-07	5.07	1.04	-4.88	SERPINB5	serpin peptidase inhibitor, clade B (ovalbumin), member 5; NULL
TC18000537.hg.1	0.000001	12.5	3.11	-4.03	NARS	asparaginyl-tRNA synthetase
TC18000047.hg.1	0.001012	4.42	1.27	-3.47	RAB31	RAB31, member RAS oncogene family
TC18000145.hg.1	0.000004	7.05	2.48	-2.85	GALNT1	UDP-N-acetyl-alpha-D-galactosamine:polypeptide N-acetylgalactosaminyltransferase 1 (GalNAc-T1); NULL
TC18000538.hg.1	0.025609	3.94	1.5	-2.64	ATP8B1	ATPase, aminophospholipid transporter, class I, type 8B, member 1; NULL
TC18000397.hg.1	0.000002	4.83	1.86	-2.6	ROCK1	Rho-associated, coiled-coil containing protein kinase 1; NULL
TC18000180.hg.1	0.000071	5.4	2.08	-2.59	ME2	malic enzyme 2, NAD(+)-dependent, mitochondrial; NULL
TC18000003.hg.1	1.73E-09	8.23	3.27	-2.51	USP14	ubiquitin specific peptidase 14 (tRNA-guanine transglycosylase)
TC18000128.hg.1	0.002544	5.54	2.31	-2.4	DSG2	desmoglein 2
TC18000480.hg.1	0.000004	4.06	1.71	-2.38	ATP5A1	ATP synthase, H+ transporting, mitochondrial F1 complex, alpha subunit 1, cardiac muscle; NULL
TC18000423.hg.1	0.000003	3.43	1.46	-2.35	SS18	synovial sarcoma translocation, chromosome 18
TC18000046.hg.1	0.000008	3.48	1.63	-2.14	RALBP1	ralA binding protein 1; NULL
TC18000998.hg.1	0.000001	3.55	1.71	-2.08	SMAD4	SMAD family member 4; NULL
TC18000069.hg.1	2.33E-09	7.06	3.42	-2.06	SEH1L	SEH1-like (S. cerevisiae)
TC18000016.hg.1	0.000754	3.18	1.57	-2.03	MYL12A	myosin, light chain 12A, regulatory, non-sarcomeric; NULL
TC18000318.hg.1	0.000041	2.74	1.39	-1.97	PPP4R1	protein phosphatase 4, regulatory subunit 1; NULL
TC18000521.hg.1	0.000058	3.81	1.93	-1.97	MBD2	methyl-CpG binding domain protein 2; NULL
TC18000373.hg.1	0.000063	2.55	1.32	-1.93	AFG3L2	AFG3 ATPase family member 3-like 2 (S. cerevisiae); AFG3 ATPase family gene 3-like 2 (S. cerevisiae); NULL
TC18000109.hg.1	6.30E-07	2.91	1.53	-1.9	IMPACT	impact RWD domain protein; NULL
TC18000461.hg.1	3.28E-08	4.7	2.54	-1.85	TPGS2	tubulin polyglutamylase complex subunit 2

TC18000501.hg.1	1.60E-07	5.71	3.11	-1.84	DYM	dymeclin; NULL
TC18000401.hg.1	0.00001	2.46	1.34	-1.84	ESCO1	establishment of sister chromatid cohesion N-acetyltransferase 1
TC18000049.hg.1	0.005038	1.72	-1.05	-1.81	VAPA	VAMP (vesicle-associated membrane protein)-associated protein A, 33kDa; NULL
TC18000052.hg.1	0.000039	2.21	1.25	-1.77	NAPG	N-ethylmaleimide-sensitive factor attachment protein, gamma
TC18000453.hg.1	0.000218	2.74	1.55	-1.77	ZNF24	zinc finger protein 24; NULL
TC18000223.hg.1	4.40E-07	2.13	1.21	-1.76	ZCCHC2	zinc finger, CCHC domain containing 2
TC18000041.hg.1	0.000005	2.08	1.19	-1.75	NDUFV2	NADH dehydrogenase (ubiquinone) flavoprotein 2, 24kDa; NULL
TC18001006.hg.1	0.000001	3.53	2.02	-1.74	PSMG2	proteasome (prosome, macropain) assembly chaperone 2; NULL
TC18000278.hg.1	0.000007	4.09	2.35	-1.74	YES1	v-yes-1 Yamaguchi sarcoma viral oncogene homolog 1
TC18000413.hg.1	0.000001	2.63	1.53	-1.72	NPC1	Niemann-Pick disease, type C1; NULL
TC18000002.hg.1	0.000029	2.29	1.34	-1.72	ROCK1P1	Rho-associated, coiled-coil containing protein kinase 1 pseudogene 1
TC18000556.hg.1	0.000005	3.89	2.28	-1.71	KDSR	3-ketodihydrosphingosine reductase; NULL
TC18000547.hg.1	2.95E-08	12.82	7.64	-1.68	LMAN1	lectin, mannose-binding, 1; NULL
TC18000094.hg.1	4.40E-07	3.14	1.87	-1.68	GATA6	GATA binding protein 6; NULL
TC18000088.hg.1	1.36E-07	4.1	2.45	-1.67	SNRPD1	small nuclear ribonucleoprotein D1 polypeptide 16kDa
TC18000557.hg.1	0.016869	1.98	1.18	-1.67	VPS4B	vacuolar protein sorting 4 homolog B (S. cerevisiae); NULL
TC18000039.hg.1	0.000037	2.15	1.3	-1.66	RAB12	RAB12, member RAS oncogene family
TC18000220.hg.1	0.002129	2.21	1.33	-1.66	KIAA1468	KIAA1468; NULL
TC18000443.hg.1	0.004757	2.22	1.34	-1.66	TRAPPC8	trafficking protein particle complex 8; NULL
TC18000221.hg.1	0.000447	2.36	1.43	-1.65	TNFRSF11A	tumor necrosis factor receptor superfamily, member 11a, NFKB activator; NULL
TC18000070.hg.1	3.68E-07	2.24	1.38	-1.63	CEP192	centrosomal protein 192kDa; NULL
TC18000496.hg.1	0.000008	2.97	1.82	-1.63	SMAD2	SMAD family member 2; NULL
TC18000175.hg.1	0.000002	2.47	1.53	-1.61	LIPG	lipase, endothelial
TC18000006.hg.1	1.43E-07	3.21	2.02	-1.58	TYMS	thymidylate synthetase; NULL
TC18000277.hg.1	0.000189	1.57	-1.01	-1.58	ENOSF1	enolase superfamily member 1; NULL

TC18000205.hg.1	0.000166	1.88	1.19	-1.57	MALT1	mucosa associated lymphoid tissue lymphoma translocation gene 1; NULL
TC18000208.hg.1	0.007673	1.71	1.09	-1.57	ZNF532	zinc finger protein 532; NULL
TC18000487.hg.1	0.000002	2.05	1.33	-1.54	PIAS2	protein inhibitor of activated STAT, 2
TC18000446.hg.1	0.002768	1.59	1.03	-1.54	GAREM	GRB2 associated, regulator of MAPK1; NULL
TC18000097.hg.1	0.000002	2.09	1.37	-1.53	RBBP8	retinoblastoma binding protein 8; microRNA 4741; NULL
TC18000156.hg.1	0.006211	1.58	1.03	-1.53	PIK3C3	phosphatidylinositol 3-kinase, catalytic subunit type 3; NULL
TC18000377.hg.1	0.000022	1.55	1.02	-1.51	PTPN2	protein tyrosine phosphatase, non-receptor type 2; NULL
TC18000090.hg.1	0.000314	2.03	1.36	-1.5	MIB1	mindbomb E3 ubiquitin protein ligase 1; NULL
TC18000532.hg.1	0.000001	2.47	1.66	-1.49	TXNL1	thioredoxin-like 1; NULL
TC18000102.hg.1	0.004441	1.64	1.14	-1.44	C18orf8	chromosome 18 open reading frame 8
TC18000507.hg.1	0.012339	2.63	1.83	-1.43	MYO5B	myosin VB
TC18000457.hg.1	2.91E-07	2.36	1.71	-1.38	RPRD1A	regulation of nuclear pre-mRNA domain containing 1A; NULL
TC18000552.hg.1	0.000005	2.8	2.02	-1.38	PIGN	phosphatidylinositol glycan anchor biosynthesis, class N
TC18000017.hg.1	0.000855	1.6	1.16	-1.38	MYL12B	myosin, light chain 12B, regulatory; NULL
TC18000114.hg.1	0.000006	2.19	1.61	-1.37	TAF4B	TAF4b RNA polymerase II, TATA box binding protein (TBP)-associated factor, 105kDa
TC18000268.hg.1	0.000009	2.44	1.78	-1.37	ADNP2	ADNP homeobox 2
TC18000574.hg.1	0.000037	2.6	1.9	-1.37	CYB5A	cytochrome b5 type A (microsomal); NULL
TC18000565.hg.1	0.000133	2.12	1.55	-1.37	TMX3	thioredoxin-related transmembrane protein 3
TC18000458.hg.1	6.79E-08	3.74	2.76	-1.35	SLC39A6	solute carrier family 39 (zinc transporter), member 6
TC18000492.hg.1	0.000538	1.74	1.3	-1.34	HDHD2	haloacid dehalogenase-like hydrolase domain containing 2
TC18000224.hg.1	0.000216	1.55	1.17	-1.33	PHLPP1	PH domain and leucine rich repeat protein phosphatase 1; NULL
TC18000148.hg.1	3.64E-07	2.86	2.17	-1.32	ELP2	elongator acetyltransferase complex subunit 2; NULL
TC18000476.hg.1	0.000022	1.53	1.16	-1.32	EPG5	ectopic P-granules autophagy protein 5 homolog (C. elegans)
TC18000272.hg.1	7.05E-08	2.41	1.84	-1.31	THOC1	THO complex 1
TC18000009.hg.1	8.28E-07	2.4	1.83	-1.31	NDC80	NDC80 kinetochore complex component; NDC80 kinetochore complex component homolog (S. cerevisiae); NULL
TC18000074.hg.1	0.000003	1.62	1.24	-1.31	RNMT	RNA (guanine-7-) methyltransferase; NULL

Supplementary Table S7. Number of CRC samples per group.

	Crh20 gain	Chr8q-gain	Chr13-gain	Chr7-gain	Chr18-loss
Selected CRC group	29	23	27	25	30
Control CRC group	16	19	19	21	16
